# Flipping the Target: Evaluating Natural LDHA Inhibitors for Selective LDHB Modulation

**DOI:** 10.3390/molecules30142923

**Published:** 2025-07-10

**Authors:** Amanda El Khoury, Christos Papaneophytou

**Affiliations:** Department of Life Sciences, School of Life and Health Sciences, University of Nicosia, 2417 Nicosia, Cyprus; amandaekhoury@gmail.com

**Keywords:** lactate dehydrogenase B, natural products, cancer metabolism, allosteric inhibition, isoform selectivity

## Abstract

Lactate dehydrogenase (LDH) catalyzes the reversible interconversion of pyruvate and lactate, coupled with the redox cycling of NADH and NAD^+^. While LDHA has been extensively studied as a therapeutic target, particularly in cancer, due to its role in the Warburg effect, LDHB remains underexplored, despite its involvement in the metabolic reprogramming of specific cancer types, including breast and lung cancers. Most known LDH inhibitors are designed against the LDHA isoform and act competitively at the active site. In contrast, LDHB exhibits distinct kinetic properties, substrate preferences, and structural features, warranting isoform-specific screening strategies. In this study, 115 natural compounds previously reported as LDHA inhibitors were systematically evaluated for LDHB inhibition using an integrated in silico and in vitro approach. Virtual screening identified 16 lead phytochemicals, among which luteolin and quercetin exhibited uncompetitive inhibition of LDHB, as demonstrated by enzyme kinetic assays. These findings were strongly supported by molecular docking analyses, which revealed that both compounds bind at an allosteric site located at the dimer interface, closely resembling the binding mode of the established LDHB uncompetitive inhibitor AXKO-0046. In contrast, comparative docking against LDHA confirmed their active-site binding and competitive inhibition, underscoring their isoform-specific behavior. Our findings highlight the necessity of assay conditions tailored to LDHB’s physiological role and demonstrate the application of a previously validated colorimetric assay for high-throughput screening. This work lays the foundation for the rational design of selective LDHB inhibitors from natural product libraries.

## 1. Introduction

Lactate dehydrogenase (LDH) is an essential metabolic enzyme responsible for the reversible conversion between pyruvate and lactate, coupled to the interconversion of NADH and NAD^+^ [1,2]. LDH exists as a tetrameric protein consisting of two distinct subunits: LDHA (muscle-type, LDH-M) and LDHB (heart-type, LDH-H), encoded by the *Ldha* and *Ldhb* genes, respectively [3]. The varying combinations of these subunits form five distinct isoforms: LDH1 (H4), LDH2 (H3M1), LDH3 (H2M2), LDH4 (H1M3), and LDH5 (M4) [4]. LDHA-rich isoforms, particularly LDH5, predominate in skeletal muscle and liver tissues and catalyze the conversion of pyruvate to lactate [2]. On the contrary, LDHB-rich isoforms, especially LDH1, are abundant in heart and brain tissues, preferentially catalyzing lactate oxidation to pyruvate, thereby linking glycolysis to the tricarboxylic acid (TCA) cycle and mitochondrial oxidative phosphorylation (OXPHOS) [5].

A hallmark of cancer cell metabolism is extensive metabolic reprogramming, widely recognized as the Warburg effect [6]. This metabolic phenotype is characterized by increased glycolytic activity and lactate production, even under aerobic conditions [7]. LDH isoforms, particularly LDHA, have been extensively studied due to their critical roles in cancer initiation, progression, metastasis, and angiogenesis. Indeed, LDHA overexpression has been documented across various malignancies, including breast, colorectal, gastric, lung, liver, brain, and bladder cancers, positioning LDHA as an important therapeutic target and prognostic biomarker [8,9].

In contrast, the biological roles and regulatory mechanisms of LDHB in cancer are relatively underexplored and poorly understood. Although typically suppressed or absent from cancers such as pancreatic, gastric, prostate, and hepatocellular carcinomas, LDHB expression is paradoxically upregulated in specific aggressive cancer subtypes. These include basal-like breast cancer (BLBC), triple-negative breast cancer (TNBC), KRAS-dependent lung adenocarcinoma, osteosarcoma, and maxillary sinus squamous cell carcinoma [10,11]. Notably, elevated LDHB expression correlates strongly with poor clinical outcomes in these malignancies. For example, LDHB is critical for sustaining tumor cell proliferation in TNBC and BLBC, where its activity significantly exceeds that observed in other breast cancer subtypes [12,13]. Similar findings have been reported in KRAS-driven lung adenocarcinoma [14] and osteosarcoma [15], where LDHB is required for tumorigenesis, proliferation, and metastatic progression.

LDHB expression is regulated through multiple oncogenic pathways. Specifically, LDHB was shown to be positively regulated by the RTK–PI3K–AKT–mTOR signaling axis and stimulated by signal transducer and activator of transcription 3 (STAT3), a key tumorigenic driver across numerous cancers [16]. Functionally, LDHB knockdown significantly impairs cell growth, proliferation, invasion, and metastasis, ultimately arresting tumor growth both in vitro and in vivo [15,17,18].

A critical mechanism underlying LDHB’s role in cancer progression involves its regulation of lysosomal function and autophagy [19]. Autophagy, a central cellular homeostatic process, initially suppresses tumor initiation but, paradoxically, facilitates tumor survival, progression, and multidrug resistance at later stages [20,21]. LDHB promotes lysosomal acidification by catalytically generating protons (H^+^), thereby sustaining lysosomal activity essential for basal autophagy. Accordingly, LDHB silencing disrupts autophagy, leads to lysosomal dysfunction, reduces tumor proliferation, and induces apoptosis via caspase-3 activation [19,22].

Moreover, LDHB plays a key role in tumor metabolic cooperation or “metabolic symbiosis”, wherein oxidative cancer cells preferentially oxidize lactate produced by glycolytic tumor cells, conserving glucose for hypoxic regions within the tumor microenvironment [23,24]. Lactate uptake by oxidative tumor cells occurs primarily through monocarboxylate transporter 1 (MCT1), after which LDHB converts lactate into pyruvate, fueling oxidative metabolism [25]. This lactate-driven metabolic symbiosis indirectly modulates glycolysis by competing with glycolytic enzymes for NAD^+^ and through the lactate-mediated inhibition of hexokinase and phosphofructokinase-1 [25,26]. Importantly, increased expression of MCT1 strongly correlates with elevated LDHB levels, particularly in TNBC [13].

Clinically, LDHB expression in breast cancer has emerged as a robust metabolic biomarker predictive of therapeutic response and prognosis. Elevated LDHB expression in BLBC and TNBC is associated with increased glycolytic dependence, higher responsiveness to neoadjuvant chemotherapy, and independently predicts pathologic complete response (pCR) irrespective of ER status and PAM50 subtype classification [12]. Conversely, high post-treatment LDHB levels in patients who fail to achieve pCR are indicative of increased proliferative potential and heightened relapse risk, suggesting its potential utility as a predictive marker in breast cancer therapy [11].

Despite compelling evidence highlighting LDHB as a promising therapeutic target, only a few selective inhibitors of this isoform have been reported. Oxamate, a structural analog of pyruvate, inhibits both LDHA and LDHB non-selectively and has demonstrated anticancer activity across various cell lines [27,28,29]. More recently, AXKO-0046 was identified as the first selective LDHB inhibitor, functioning through an uncompetitive mechanism by binding to a unique allosteric site distinct from the catalytic domain [30]. However, the clinical relevance of AXKO-0046 and similar compounds still requires extensive validation. Our group previously identified two FDA-approved anticancer drugs, tucatinib and capmatinib, originally developed for the treatment of breast and lung cancers, as uncompetitive inhibitors of LDHB. They exert their inhibitory effect by binding to an allosteric site distinct from the enzyme’s catalytic domain [31]. However, the primary aim of that study was to validate the high-throughput colorimetric assay developed by our group [32] for detecting LDHB inhibitors, rather than to identify novel inhibitors. As a result, the discovery of selective LDHB inhibitors remains limited. Therefore, there is an urgent need for alternative strategies to identify potent and isoform-specific LDHB inhibitors to support therapeutic development.

Natural products, particularly phytochemicals, represent a promising but underexplored resource in this regard. Historically, phytochemicals have significantly contributed to anticancer therapy, with nearly 25% of currently approved anticancer drugs being derived from plant-based sources [33,34]. Despite advances in synthetic drug development, phytochemicals remain highly valuable due to their structural diversity, bioactivity, and therapeutic potential. Notable examples include vinblastine, paclitaxel, and camptothecin [35]. Additionally, phytochemical-derived compounds often enhance the efficacy of conventional treatments through synergistic effects, including reduced relapse, drug resistance, and metastasis [36].

Despite increasing interest in LDHA as a therapeutic target, using both small molecules [37] and natural products [38], the systematic screening of inhibitors that specifically target LDHB remains limited. In contrast, recent research efforts have predominantly focused on LDHA, leading to the identification of numerous natural inhibitors with diverse chemical scaffolds and pharmacological properties (reviewed in [39]). Several natural compounds have been shown to inhibit LDH broadly; however, isoform-specific targeting remains largely unexplored. For example, gossypol, a natural compound derived from cottonseed, has been characterized as a non-selective LDH inhibitor, exhibiting Ki values of 1.9 mM, 1.4 mM, and 4.2 mM for LDHA, LDHB, and LDHC, respectively [40,41,42]. However, gossypol has demonstrated limited clinical efficacy in trials involving metastatic adrenal cancer, malignant glioma, and chemotherapy-resistant metastatic breast cancer [43]. Thus, identifying phytochemicals with inhibitory activity against LDHB may yield innovative strategies for cancer therapy and other LDHB-related pathologies, such as cardiovascular, neurodegenerative, and chronic liver or kidney diseases [38].

Furthermore, natural products such as flavonoids, alkaloids, and terpenoids are known to inhibit LDHA effectively, and many have shown potential in anticancer therapy [38]. These compounds typically act through competitive or covalent mechanisms, targeting the enzyme’s active site or NADH-binding region. The most frequently observed mechanism entails direct occupation of the catalytic pocket, thereby preventing the conversion of pyruvate to lactate [44,45]. In contrast, as mentioned above, recent studies have suggested that LDHB can be inhibited allosterically by small molecules. However, the mechanisms by which natural compounds inhibit LDHB remain largely unexplored.

Another factor hindering the discovery of LDHB inhibitors is a recurring methodological limitation in the literature. Although LDHB is occasionally included in comparative LDH studies, its enzymatic activity is frequently assessed under conditions optimized for LDHA, namely, using the pyruvate-to-lactate reaction and monitoring NADH consumption. However, LDHB naturally catalyzes the reverse reaction, converting lactate to pyruvate using NAD^+^ as a cofactor [46]. This experimental mismatch may mask the true inhibitory effects of certain compounds on LDHB.

To address the above limitations, we combined structure-based virtual screening with isoform-specific enzymatic assays to identify and characterize natural compounds with potential selectivity toward LDHB. Our aim was to evaluate whether phytochemicals previously reported as LDHA inhibitors—or as general LDH inhibitors—also inhibit LDHB, and to determine their selectivity and mechanisms of action under biologically relevant conditions.

## 2. Results

### 2.1. Searching for Natural LDHB Inhibitors

#### 2.1.1. Constructing a Virtual Library of Natural Compounds

Virtual screening and molecular docking are powerful computational methods used to identify novel enzyme inhibitors efficiently [47,48]. Integrating these computational approaches with experimental validation significantly enhances drug discovery, accelerating the identification and development of promising therapeutic candidates [49].

Our study began with a systematic review of the literature to identify natural compounds previously evaluated as LDH/LDHA inhibitors. This effort yielded seven key studies (summarized in Table 1), which collectively represent significant advances in the characterization of natural LDH inhibitors. From these sources, we compiled a library of 115 natural compounds (Supplementary Table S1), all of which were included in our subsequent analyses regardless of their previously reported potency. As discussed earlier, while many natural products have demonstrated general LDH inhibitory activity, isoform-specific targeting—particularly of LDHB—remains largely underexplored. This study aimed to address that gap by applying isoform-specific approaches to identify selective natural inhibitors of LDHB.

#### 2.1.2. Molecular Docking

While monomeric forms are often used in docking studies for computational efficiency, LDHB functions biologically as a tetramer, making its quaternary structure essential for accurate modeling. Based on our hypothesis that natural compounds may inhibit LDHB through an allosteric mechanism, we selected the tetrameric structure of LDHB (PDB ID: 7DBJ) [30]. This structure was chosen because it includes oxamate, NADH, and AXKO-0046, allowing for the precise identification of binding-site coordinates. Notably, oxamate binds to the active site within each of the four subunits, while AXKO-0046 binds allosterically at the interface between adjacent subunits, specifically between chains A and C, as well as between chains B and D in the 7DBJ structure.

Initial docking studies targeted both the active site within chains A and C and the allosteric site located at their interface. However, upon observing that all tested compounds predominantly localized near the subunit interface, similar to AXKO-0046 [30], a subsequent redocking was performed using the precise coordinates of AXKO-0046 within the 7DBJ structure to better evaluate their binding affinities at the allosteric site. AXKO-0046 served as the control compound for comparative analysis throughout the study.

**Figure 1:**
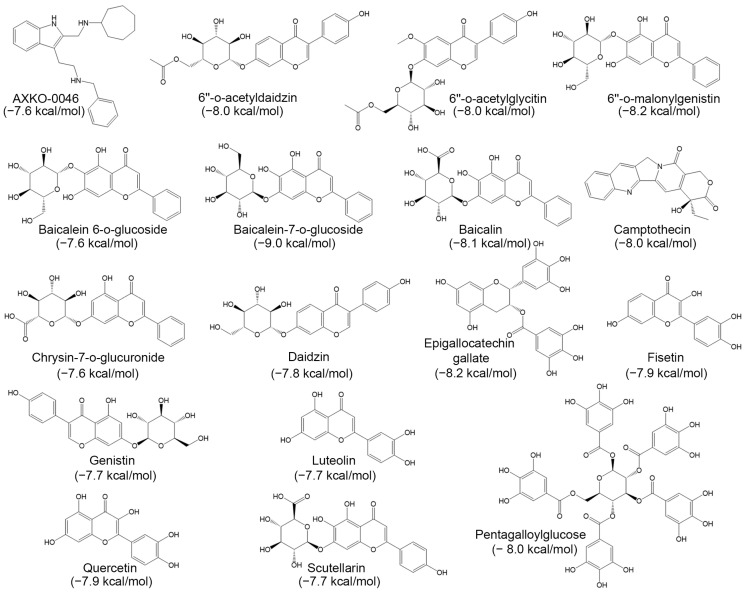
The top-scoring natural compounds identified through molecular docking as potential allosteric inhibitors of LDHB. Docking was conducted using the crystal structure of LDHB (PDB ID: 7DBJ). The chemical structures and corresponding docking scores (in kcal/mol) of the 16 compounds that achieved scores equal to or better than that of AXKO-0046 (−7.6 kcal/mol), a known uncompetitive inhibitor of LDHB, are illustrated.

The control compound, AXKO-0046, achieved a docking score of −7.6 kcal/mol. Of the 115 tested compounds (Supplementary Table S1), 16 scored equal to or better than the control (Figure 1), while 99 were excluded due to lower binding affinities. The highest score was observed for baicalein-7-*O*-glucoside (−9.0 kcal/mol), with other top compounds scoring between −7.6 and −8.2 kcal/mol.

#### 2.1.3. Drug-Likeness Analysis

To identify suitable candidates for LDHB inhibition, the top 16 phytochemicals identified by molecular docking were further evaluated for their drug-likeness and pharmacokinetic properties using the SwissADME platform, which assesses key absorption, distribution, metabolism, and excretion (ADME) parameters. The compounds were ranked based on their key pharmacokinetic parameters, including gastrointestinal (GI) absorption, compliance with established drug-likeness rules (Lipinski, Veber, Egan, and Muegge), absence of Pan-Assay INterference Compounds (PAINS) and Brenk structural alerts, and adherence to lead-likeness criteria.

Among the screened compounds, only four, i.e., camptothecin (−8.0 kcal/mol), fisetin (−7.9 kcal/mol), luteolin (−7.7 kcal/mol), and quercetin (−7.9 kcal/mol), demonstrated favorable ADME profiles and were selected for further molecular docking and in vitro analyses. All compounds demonstrated high predicted GI absorption and satisfied major drug-likeness criteria, including Lipinski’s and Veber’s rules, with no rule violations. Camptothecin showed the highest lipophilicity and the lowest water solubility among the group, while the flavonoids (quercetin, fisetin, and luteolin) exhibited good solubility and favorable lipophilic balance. Although the latter compounds contained catechol groups associated with structural alerts (PAINS) due to the presence of catechol groups, their overall ADME profiles were deemed acceptable, supporting their prioritization for molecular docking and further in silico analysis (Table 2).

In contrast, the remaining compounds were excluded from further investigation due to their suboptimal pharmacokinetic characteristics, including low GI absorption, multiple rule violations, and/or the presence of structural alerts. Such features are associated with reduced bioavailability, increased toxicity risk, and a higher likelihood of false positives in bioassays, thereby limiting their drug development potential.

The toxicological properties of the selected phytochemicals were evaluated using two independent prediction servers—ProTox 3.0 and StopTox—to ensure the reliability and consistency of the results. Both platforms provided concordant and satisfactory toxicity profiles for all four candidate compounds.

According to ProTox 3.0 (Table 3), all compounds were predicted to be inactive for hepatotoxicity, with high confidence scores (e.g., 0.83 for camptothecin and 0.70 for fisetin). Neurotoxicity and nephrotoxicity were flagged for some compounds: camptothecin was predicted to be active for both endpoints, while luteolin and quercetin showed potential nephrotoxicity (probability 0.62) and fisetin showed a similar trend. Toxicity classification based on oral LD_50_ values revealed that camptothecin, fisetin, and quercetin belong to toxicity class 3, with predicted LD_50_ values of 50 mg/kg (camptothecin, 100% accuracy) and 159 mg/kg (fisetin and quercetin, with 72.9% and 100% accuracy, respectively). In contrast, luteolin was assigned to toxicity class 5 with a substantially higher predicted LD_50_ of 3919 mg/kg (accuracy: 70.97%), indicating a lower acute toxicity potential.

Further screening using the StopTox platform (Table 4) evaluated toxicity across six endpoints, including acute oral, dermal, and inhalation toxicity, skin and eye irritation/corrosion, and skin sensitization. All four compounds fulfilled most of the safety criteria, although minor discrepancies were observed in skin-related endpoints.

Collectively, these results suggest that all the selected phytochemicals exhibit acceptable toxicity profiles, with luteolin showing the lowest predicted toxicity overall. Camptothecin’s predicted neurotoxicity and lower LD_50_ value warrant consideration in future in vivo validations.

Subsequently, the inhibitory effects of these four compounds were assessed through in vitro experimentation.

### 2.2. In Vitro Evaluation of Potential LDHB Inhibitors

We evaluated the inhibitory effects of the four top-ranked candidate compounds using a recently developed and validated, by our team, in vitro colorimetric assay in a 96-well format [31,32]. This assay is based on the detection of NADH generated during the enzymatic conversion of lactate to pyruvate (via reduction of NAD^+^), which subsequently reacts with nitro blue tetrazolium (NBT) in the presence of phenazine methosulfate (PMS). This reaction produces a blue-purple formazan dye with a maximum absorbance at approximately 570 nm.

Given that most studies report LDHA inhibition by natural compounds in the micromolar range [39], our initial objective was to determine whether the selected compounds, at concentrations ranging from 5 to 100 µM, interfered with formazan formation. Our findings indicated that none of the four candidate compounds interfered with the assay readout at the maximum tested concentration of 100 μΜ (Supplementary Figure S1), thereby validating the system’s reliability for accurately assessing LDHB activity.

**Figure 2:**
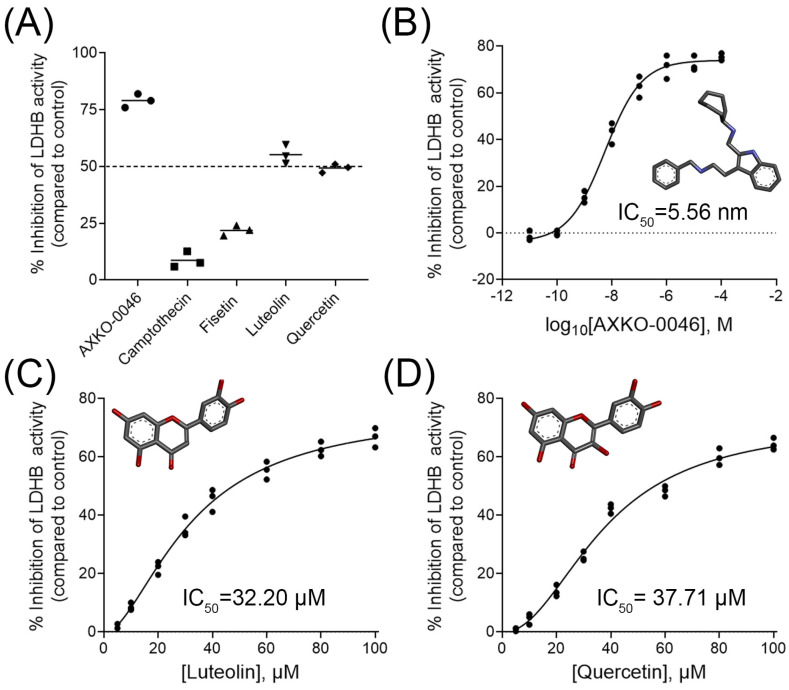
The evaluation of the top candidate compounds for LDHB inhibition. The inhibitory activity of the selected compounds was assessed using a 96-well endpoint colorimetric assay, as described in the Section 4. (**A**) The inhibition of LDHB activity by the top four candidates identified from virtual screening, compared to the known LDHB inhibitor AXKO-0046. The horizontal bars indicate the mean values from three independent experiments. The dashed line indicates 50% inhibition. Dose–response curves show the inhibitory effect of AXKO-0046 (**B**), luteolin (**C**), and quercetin (**D**) on LDHB activity. The percentage of LDHB inhibition was determined by comparing the enzyme activity in the presence of each compound to that of a control without any of the inhibitors. IC_50_ values were calculated using nonlinear regression analysis. The molecular structures of the compounds are shown in each panel.

We then examined the inhibitory effect of each compound at a concentration of 50 µM on LDHB activity (Figure 2). As expected, AXKO-0046 significantly inhibited LDH-B activity by approximately 75% (Figure 2A), consistent with its previously reported potency [30]. Interestingly, camptothecin—despite having the highest virtual screening score—exhibited only minimal inhibition. This limited effect may be attributed to its high lipophilicity, as indicated by the ADME analysis (Table 2). Among the three flavonoids tested, luteolin showed the strongest inhibition (~55%), followed by quercetin (~50%), while fisetin exhibited only moderate activity (~22%) and was therefore excluded from further analysis. Given that both luteolin and quercetin inhibited LDHB activity by approximately 50%, we proceeded to assess their inhibitory potency through dose–response analyses.

As anticipated, AXKO-0046 exhibited a strong, dose-dependent inhibitory effect on LDHB, with an IC_50_ value of 5.56 nM (Figure 2B), in line with previous studies [30,31]. Notably, luteolin and quercetin also demonstrated dose-dependent inhibition, albeit with considerably higher IC_50_ values of 32.20 µM and 37.71 µM, respectively (Figure 2C,D). These findings suggest that while luteolin and quercetin are substantially less potent than AXKO-0046, they may serve as modest yet biologically relevant natural LDHB inhibitors.

### 2.3. Kinetic Studies

We subsequently carried out substrate competition assays to clarify the mechanisms by which luteolin and quercetin inhibit LDHB. The inhibitory effects of these compounds were evaluated across varying concentrations of lactate and NAD^+^, revealing insights into their modes of action. Michaelis–Menten analysis demonstrated that both compounds likely exert their inhibitory effects through an uncompetitive inhibition mechanism. Specifically, increasing concentrations of the inhibitors resulted in a concurrent decrease in both Km and Vmax values for LDHB (Table 5). We then investigated whether a time-dependent effect existed on the onset of inhibition by adjusting the duration for which luteolin, or quercetin, and LDHB were pre-incubated before initiating the enzymatic reaction. Notably, when LDHB was pre-incubated with both compounds for 120 min, the inhibitory effect did not increase with longer pre-incubation times (Supplementary Figure S2).

**Figure 3:**
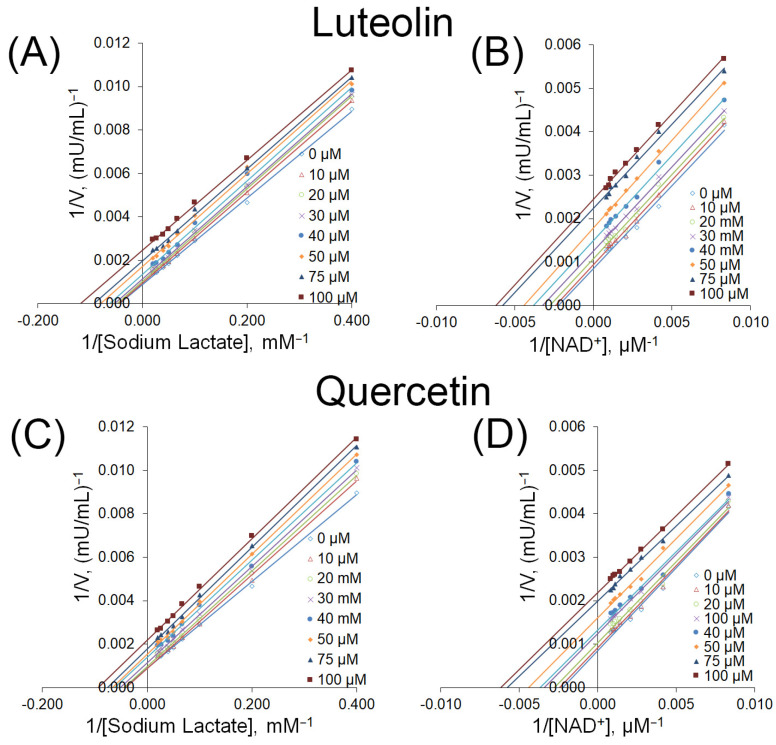
A kinetic analysis of LDHB inhibition by luteolin and quercetin. The inhibitory effects of luteolin (**A**,**B**) and quercetin (**C**,**D**) on LDHB activity were assessed using varying concentrations of sodium lactate (**A**,**C**) and NAD^+^ (**B**,**D**). Lineweaver–Burk plots were constructed using linear regression analysis from the kinetic data (see Table 2), with each curve corresponding to a different inhibitor concentration (0–100 μM).

Furthermore, Lineweaver–Burk plots displayed nearly parallel lines (Figure 3), supporting the conclusion of uncompetitive inhibition, wherein the inhibitors bind exclusively to the enzyme–substrate complex.

Subsequently, we utilized mixed-model inhibition analysis to determine the mechanism through which LDHB is inhibited by luteolin and quercetin. The results suggest that both compounds predominantly interact with the enzyme–substrate complex, indicating an uncompetitive inhibition mechanism rather than direct competition at the active site with either lactate or NAD^+^. This conclusion is supported by the calculated α values, which were less than 1 and indicative of uncompetitive inhibition. (Table 6). However, additional investigations are necessary to further clarify the specific inhibitory processes involved.

### 2.4. Molecular Interaction Analysis

**Figure 4:**
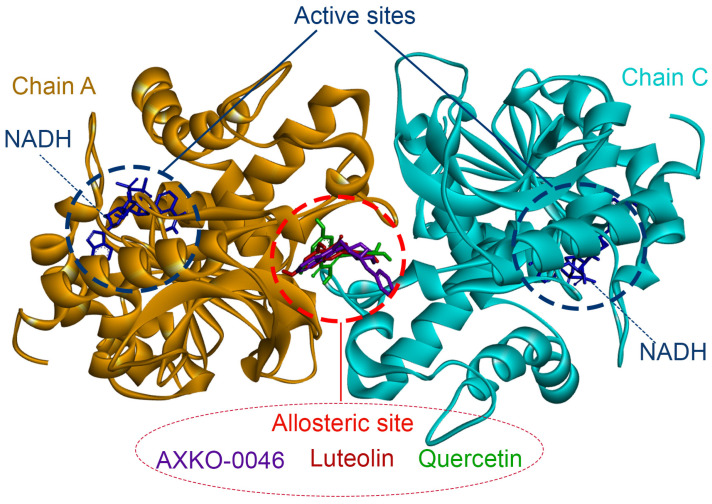
The predicted binding of AXKO-0046, luteolin, and quercetin on LDHB. Structural superposition shows LDHB in complex with NADH (blue sticks) bound at the active sites of each monomer (chain A: orange; chain C: cyan). AXKO-0046 (purple), luteolin (red), and quercetin (green) are all localized at the allosteric site formed at the dimer interface, distal to the catalytic site. This binding mode is consistent with their experimentally determined inhibition behavior, supporting an uncompetitive mechanism of action.

Molecular docking and interaction analyses between LDHB and the top candidate compounds were conducted using BIOVIA Discovery Studio Visualizer 2025 (Dassault Système, San Diego, CA, USA) to further elucidate their binding characteristics. As shown in Figure 4, both luteolin and quercetin bind to the same allosteric site located at the dimer interface between chains A and C of LDHB (PDB ID: 7DBJ), positioned near to but distinct from the active sites where NADH/NAD^+^ and pyruvate/lactate bind. Notably, this binding site coincides with that of the well-characterized uncompetitive inhibitor AXKO-0046. These structural findings are consistent with our in vitro kinetic data, which suggested that both luteolin and quercetin act through an allosteric mechanism of inhibition.

**Figure 5:**
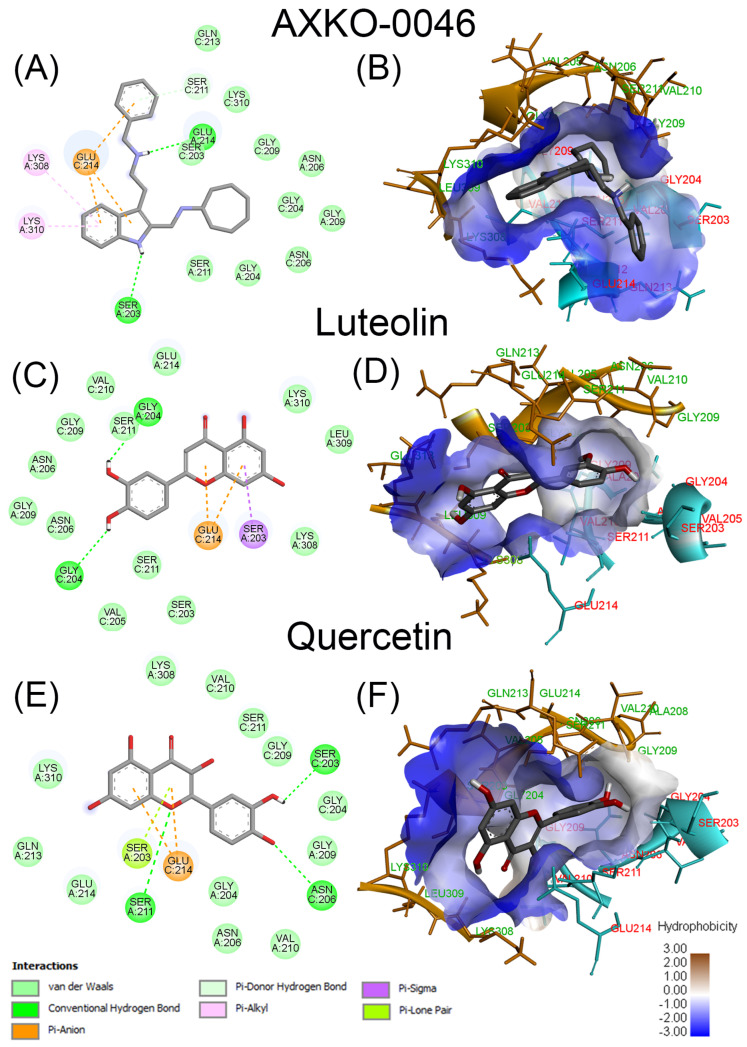
The molecular interactions of AXKO-0046 (**A**,**B**), luteolin (**C**,**D**), and quercetin (**E**,**F**) with the allosteric site of LDHB located in the interphase of chains A and C (in 7DBJ), illustrated as 2D interaction diagrams (left panels) and 3D hydrophobic surface maps (right panels). In the 2D diagrams, the amino acid residues from LDHB chains A and C involved in the interactions are labeled and highlighted with colored circles. These circles correspond to different interaction types, as indicated in the interaction key located in the bottom left panel of the figure. In the 3D hydrophobic surface maps, key hydrophobic (brown) and hydrophilic (blue) regions of the binding pocket are shown, along with hydrogen bonds and other non-covalent interactions. The color gradient reflects surface hydrophobicity, as explained in the key shown in the bottom right panel of the figure. The docking studies were carried out using PyRx v1.1 (AutoDock Vina). The 2D and 3D images were obtained using BIOVIA Discovery Studio.

Figure 5 illustrates the predicted molecular interactions of AXKO-0046, luteolin, and quercetin at the allosteric site of LDHB, located at the interface between chains A and C. The figure includes 2D interaction diagrams (Figure 5A,C,E) and corresponding 3D hydrophobic surface maps (Figure 5B,D,F), which together illustrate the binding modes and physicochemical environments of each ligand. The allosteric pocket is defined by a shallow cavity composed mainly of hydrophobic residues—such as Gly204, Asn206, Gly209, Ser211, and Lys308/310—interspersed with key polar residues that contribute to binding specificity. Hydrophobic surface mapping highlights this dual character: while van der Waals and aromatic interactions (π–π, π–alkyl) dominate the binding interface, specific hydrogen bonds play a crucial role in stabilizing ligand orientation [30].

In detail, AXKO-0046 exhibited the most extensive interaction profile among the compounds tested (Figure 5A,B). It formed key polar interactions, including a hydrogen bond between its indole NH group and the carbonyl oxygen of Ser203, and additional hydrogen bonds involving its amino groups and the carboxyl side chain of Glu214. These polar contacts are reinforced by a dense network of hydrophobic interactions—primarily van der Waals forces and aromatic (π–π and π–alkyl) interactions—with surrounding residues such as Gly204, Gly209, and Lys308/310. The 3D hydrophobic surface map confirms that AXKO-0046 spans both polar (blue) and nonpolar (brown) regions within the allosteric pocket, consistent with its high binding affinity and specificity. This combination of hydrophobic anchoring and polar recognition likely underlies its potent allosteric inhibition of LDHB. Our findings align closely with the observed interaction of AXKO-0046 with the residues of LDHB forming the allosteric site, as revealed by its crystal structure with the inhibitor [30].

On the other hand, luteolin and quercetin showed more moderate interaction profiles, as illustrated by the 2D interaction diagrams (Figure 5C,E, respectively). Both flavonoids formed hydrogen bonds with key polar residues, including Ser203 and Glu214, like AXKO-0046. However, the number and strength of these polar contacts were less extensive. Luteolin established fewer interactions overall, relying mainly on van der Waals forces and limited π–π interactions to stabilize its binding (Figure 5C). Quercetin, by contrast, formed slightly more hydrogen bonds—likely due to its additional hydroxyl group—enhancing its polar interaction network (Figure 5E).

The 3D hydrophobic surface maps further support these findings: both luteolin (Figure 5D) and quercetin (Figure 5F) occupy similar regions within the allosteric pocket as AXKO-0046, but with reduced surface complementarity and shallower engagement in hydrophobic zones. These results suggest that, while luteolin and quercetin are capable of binding to the LDHB allosteric site, their lower affinity reflects a suboptimal combination of hydrophobic anchoring and polar recognition. Nevertheless, their natural origin and favorable pharmacological properties make them attractive scaffolds for further optimization.

## 3. Discussion

Natural LDH inhibitors exhibit structural diversity and multifunctionality, making them strong candidates for multitarget therapies in cancer. Several studies have highlighted the potential of natural compounds as LDHA inhibitors. For instance, Li et al. [51] examine the bioactivity screening, extraction, and separation of lactate dehydrogenase inhibitors from *Polygala tenuifolia* using a hyphenated strategy. Their research identifies five promising lactate dehydrogenase inhibitors—sibiricose A5, 3,6′-di-*O*-sinapoyl-sucrose, glomeratose A, tenuifoliside B, and tenuifoliside C—and successfully isolates them with high purity using microwave-assisted extraction coupled with countercurrent chromatography. The bioactivity of these compounds was confirmed through testing with PC12 cells, supporting their potential use in ischemic stroke treatment. Ahmet et al. [45] investigated the anticancer potential of phytochemicals derived from *Oroxylum indicum* targeting LDHA using a comprehensive bioinformatic approach. The study screened 52 phytochemicals via molecular docking, ADME/T analysis, molecular dynamics simulation, and MM/GBSA free binding energy calculations. Three lead compounds—chrysin-7-*O*-glucuronide, oroxindin, and oroxin A—demonstrated favorable binding affinities (−8.2 to −8.0 kcal/mol), pharmacokinetic profiles, and conformational stability. Oroxindin exhibited the most favorable binding free energy (−46.47 kcal/mol), surpassing the control drug Sunitinib. However, the clinical translation of phytochemicals is hindered by several limitations, including low solubility, toxicity (e.g., gossypol), and insufficient in vivo validation. While substantial efforts have been devoted to identifying LDHA inhibitors, studies specifically targeting LDHB with natural or small-molecule inhibitors remain limited [42].

In this study, we retrieved 115 natural compounds previously identified as LDHA inhibitors from the literature (Table 1 and Supplementary Table S1) and evaluated their potential to inhibit LDHB. The research studies included in Table 1 indicate that natural compounds typically inhibit LDHA through competitive mechanisms. Interestingly, LDHA can also be inhibited by small molecules, such as (R)-GNE-140, which competitively target both the substrate- and cofactor-binding sites [53]. Other known small-molecule competitive inhibitors of LDHA include oxamate (a pyruvate competitive inhibitor), FX11 (an NADH-competitive inhibitor), and NHI (which is competitive against both pyruvate and NADH) [37]. It is important to note that LDHA can be inhibited through mechanisms beyond competitive inhibition. Friberg et al. [54] demonstrated that phthalimide and dibenzofuran selectively inhibit LDHA by binding to an allosteric site distinct from the enzyme’s active site. As discussed above, recent findings regarding selective LDHB inhibition reported by Shibata et al. [30] and the initial docking studies conducted in this study suggest that LDHB is inhibited through an allosteric mechanism, as further discussed below. To support our analyses, we utilized AXKO-0046, a well-characterized and selective uncompetitive inhibitor of LDHB, as a control compound.

Virtual screening of the 115 compounds revealed 16 candidates with docking scores equal to or more favorable (i.e., more negative) than that of AXKO-0046 (−7.6 kcal/mol) (Figure 1). ADME/T profiling (Table 2 and Table 3) of these 16 phytochemicals highlighted four top candidates: camptothecin (−8.0 kcal/mol), fisetin (−7.9 kcal/mol), luteolin (−7.7 kcal/mol), and quercetin (−7.9 kcal/mol). While these docking scores suggested strong potential for interaction with LDHB, in vitro enzymatic assays revealed that camptothecin and fisetin had only minor effects on LDHB activity (Figure 2). This discrepancy may be attributed to pharmacokinetic limitations such as low solubility, poor permeability, or high lipophilicity, despite their favorable docking affinities. In contrast, luteolin and quercetin demonstrated concentration-dependent inhibition of LDHB, with IC_50_ values of 32.20 μM and 37.71 μM, respectively (Figure 2). These values are within the range of activity reported for various natural LDH inhibitors [44,50] and suggest that both flavonoids hold promise as functional modulators of LDHA and LDHB activity (discussed further below).

To gain insights into their inhibitory mechanism, we performed detailed kinetic analyses (Table 5 and Figure 3). Both luteolin and quercetin displayed inhibition patterns consistent with uncompetitive inhibition, where the inhibitor binds only to the enzyme–substrate complex. This contrasts with the classical competitive inhibition observed for many LDHA inhibitors, which compete with NADH or pyruvate for the active site. Our mixed-model inhibition analysis further supported this mechanism, with α values less than 1, a hallmark of uncompetitive inhibition (Table 6). These findings are consistent with the behavior of AXKO-0046 and support the notion that LDHB is amenable to allosteric regulation.

Molecular docking using the tetrameric structure of LDHB (PDB ID: 7DBJ) confirmed that both luteolin and quercetin bind at the allosteric site located at the interface between chains A and C—the same region targeted by AXKO-0046 (Figure 4). Their binding is primarily stabilized through van der Waals interactions but also involves hydrogen bonds and π–π or π–π-alkyl interactions with key residues such as Ser203, Glu214, Gly204, Asn206, and Lys308/310. These interactions are visualized in the 2D interaction diagrams and 3D hydrophobic surface representations (Figure 5), which illustrate the dual hydrophobic–hydrophilic nature of the allosteric pocket. Notably, the hydrophobic cavity anchors the inhibitors, while key polar residues ensure specificity and orientation.

The structural and kinetic congruence between luteolin/quercetin and AXKO-0046 provide strong support for the concept that natural flavonoids can function as allosteric LDHB inhibitors. This offers an alternative to classical competitive inhibition and opens new avenues for the development of isoform-selective LDH modulators. Given LDHB’s emerging role in cancer metabolism, particularly in tissues with high oxidative demands or in tumors that switch from LDHA to LDHB expression, selective LDHB inhibition may provide unique therapeutic advantages. Together, our findings establish luteolin and quercetin as natural, biologically relevant uncompetitive inhibitors of LDHB and highlight their potential as lead compounds for the development of allosteric modulators targeting this enzyme. These insights pave the way for further structural optimization and in vivo validation in cancer and metabolic disease models.

Notably, luteolin and quercetin have previously been identified as competitive inhibitors of LDHA. For instance, Li et al. [50] reported that both compounds inhibited LDHA activity, with inhibition rates of 50.7% and 53.6%, respectively, using electrophoretically mediated microanalysis (EMMA). Interestingly, and consistent with our findings on LDHB, camptothecin showed no inhibitory effect on LDHA in that study. In a more recent report, Yırtıcı [44] identified fisetin, luteolin, and quercetin as LDHA inhibitors through enzymatic assays. Among them, fisetin exhibited the strongest inhibition with an exceptionally low IC_50_ value of 0.066 μM, followed by luteolin (IC_50_ = 1.792 μM), while quercetin showed moderate activity (IC_50_ = 33.647 μM). Yırtıcı [44] further explored the molecular interactions of luteolin and quercetin with LDHA using docking simulations. Both flavonoids were found to bind within the active site, forming key hydrogen bonds and hydrophobic interactions with catalytically important residues. Specifically, luteolin established stable hydrogen bonds with Arg105 and Asp168, while quercetin interacted with His193 and Asn138, residues critical for NADH binding and enzymatic function. These results confirm their mode of action as competitive inhibitors of LDHA.

**Figure 6:**
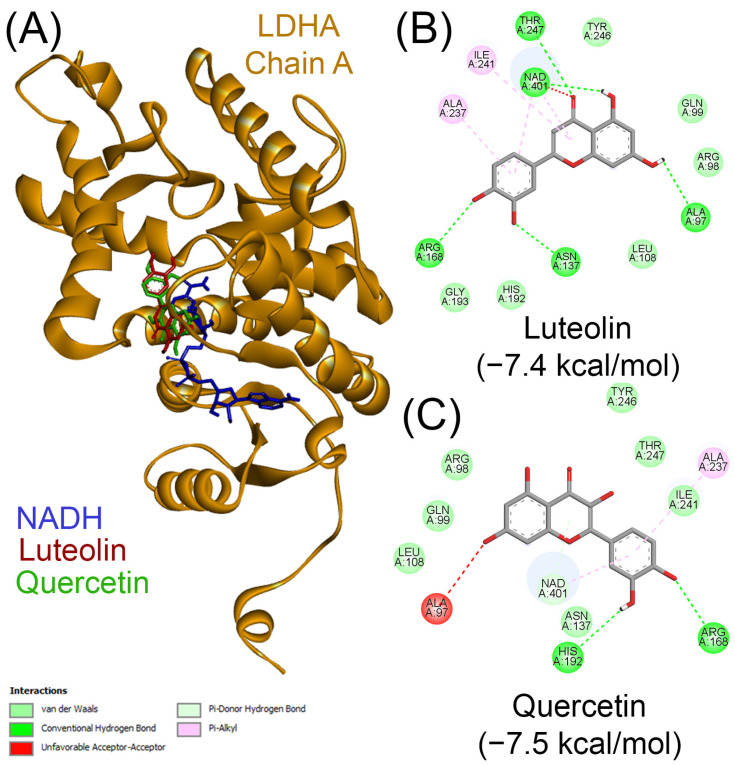
The docking of luteolin and quercetin with human LDHA. (**A**) The overall structure of LDHA (PDB ID: 4ZVV; chain A), showing the binding orientations of NADH (blue), luteolin (red), and quercetin (green) within the active site. The molecular interactions of luteolin (**B**) and quercetin (**C**) with LDHA are illustrated as 2D interaction diagrams. In these diagrams, interacting amino acid residues from LDHA chain A are labeled and highlighted with colored circles, corresponding to the different interaction types indicated in the interaction key in the bottom left panel. In (**B**,**C**), the binding scores are indicated in parentheses as kcal/mol.

To further investigate the isoform-specific inhibitory behavior of flavonoids, we conducted comparative molecular docking of luteolin and quercetin against human LDHA using the crystal structure with PDB ID 4ZVV (chain A), which is co-crystallized with the potent active-site inhibitor (R)-GNE-140. Docking was performed using the active-site coordinates described by Shu et al. [55]. In agreement with previous reports, our docking results revealed that both luteolin and quercetin bind within the active site of LDHA, in close proximity to NADH (Figure 6A). Luteolin exhibited a binding score of −7.5 kcal/mol and formed several key hydrogen bonds with residues Arg168, Gly193, His192, Asn137, and Thr247, along with coordination near the NADH cofactor (Figure 6B). Quercetin showed a similar binding profile, with a score of −7.4 kcal/mol, forming hydrogen bonds with His192, Arg168, and Asn137, as well as engaging in hydrophobic interactions with surrounding residues (Figure 6C). These findings further support the classification of luteolin and quercetin as competitive inhibitors of LDHA. This divergence in binding mechanism between LDHA and LDHB underscores the isoform-specific behavior of flavonoids and highlights the importance of empirical validation when repurposing inhibitors across LDH isoforms. Together, these findings underscore the importance of structure-guided selectivity assessments and provide a framework for the rational design of dual or isoform-selective LDH inhibitors with potential therapeutic implications.

These findings highlight a key challenge in developing LDH inhibitors, particularly in distinguishing and selectively targeting the LDHA and LDHB isoforms. Historically, LDHA and LDHB have been considered functionally similar enzymes because they both catalyze the reversible interconversion of pyruvate and lactate while facilitating the redox cycling of NADH and NAD^+^. This assumption was supported by evidence showing that the enzymatic activity of purified LDH-5 (composed of four LDHA subunits) closely resembles that of LDH-1 (composed of four LDHB subunits) under physiological conditions [56]. Additionally, zymogram analysis has demonstrated that both isoforms can utilize pyruvate and lactate as substrates [57], indicating their comparable efficiency in catalyzing the conversion of pyruvate to lactate [58].

However, LDHA and LDHB differ significantly in substrate preference and physiological roles. LDHA has a higher affinity for pyruvate, primarily converting it to lactate while oxidizing NADH to NAD^+^, whereas LDHB favors lactate, converting it to pyruvate while reducing NAD^+^ to NADH [58,59]. This metabolic distinction is reflected in their tissue distribution: LDHA predominates in glycolytic tissues such as skeletal muscle, while LDHB is enriched in oxidative tissues like the heart. Additionally, LDHB exhibits substrate inhibition at elevated pyruvate concentrations [29]. At the molecular level, LDHA carries a net negative charge (−6), while LDHB is slightly basic (+1). The functional behavior of LDH tetramers is influenced by the relative composition of the LDHA and LDHB subunits; LDH-5 (LDHA_4_) favors lactate production, whereas LDH-1 (LDHB_4_) facilitates lactate oxidation [60].

LDHB is composed of four identical subunits, each consisting of 334 amino acids and organized into two major structural domains. The NAD^+^-binding domain (residues 21–163 and 249–267) adopts a characteristic Rossmann fold that accommodates the cofactor, while the substrate-binding domain (residues 164–248 and 268–332) forms the pocket that coordinates pyruvate or lactate. While the overall architecture closely resembles that of LDHA, key residue-level differences distinguish the isoforms. For example, His192, Asp165, Arg168, and Thr247 in LDHA correspond to His193, Asp166, Arg169, and Thr248 in LDHB, respectively. These subtle yet functionally important substitutions can influence substrate orientation and inhibitor binding, as shown in previous comparative structural studies [5,61].

**Figure 7:**
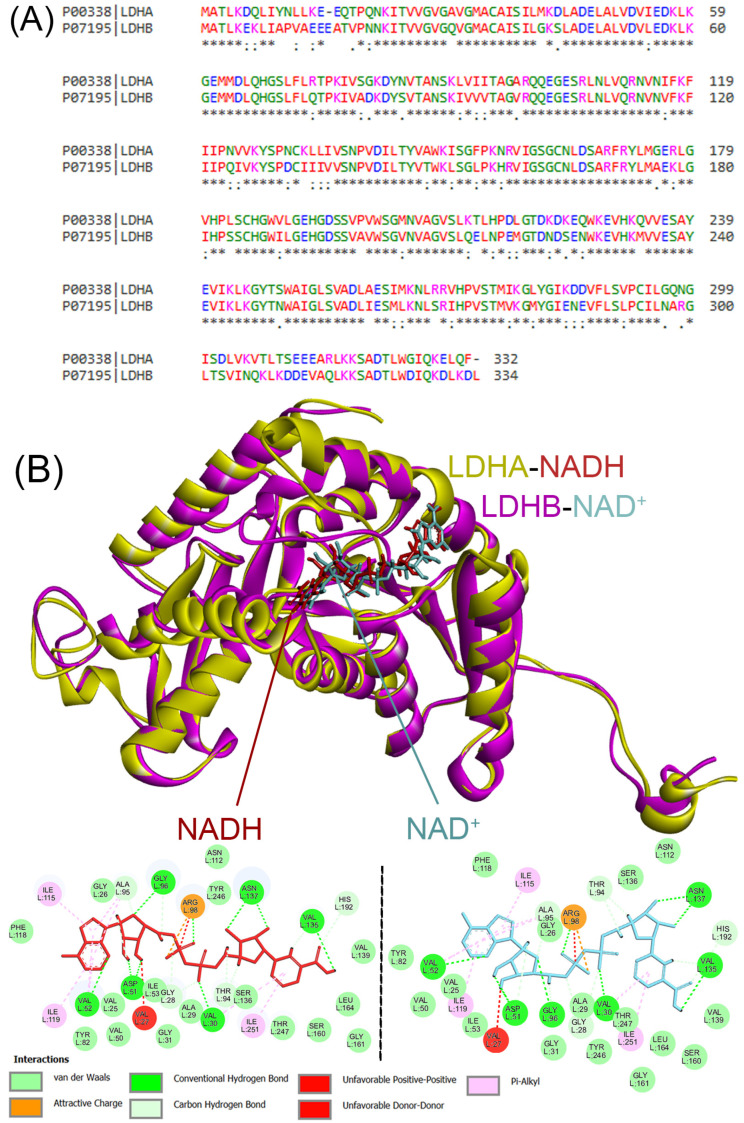
(**A**) The pairwise sequence alignment of human LDHA and LDHB. Identical amino acids are marked with an asterisk (*), conserved amino acids with a colon (:), and semi-conserved amino acids with a period (.). The residues are color-coded based on their chemical properties: acidic (red), basic (blue), polar (cyan), hydrophobic (green), and others (magenta). The alignment shows 75.1% identity (251 out of 334 residues) and 88.6% similarity (296 out of 334 residues), with notable substitutions near the active site and cofactor-binding regions. (**B**) Structural superposition of human LDHA (PDB ID: 4zvv; chain A, gold) bound to NADH (red) and LDHB (PDB ID: 1t2f; chain A, purple) bound to NAD^+^ (cyan), generated using UCSF ChimeraX v 1.9. Despite the overall structural conservation, differences are evident in the substrate-binding pockets and NADH/NAD^+^ interaction sites, highlighted in the two-dimensional interaction diagrams below the structure. Critical residues participating in hydrogen bonding and hydrophobic interactions are depicted, and ligand interaction types are labeled according to their interaction characteristics. These localized variations may influence isoform-specific ligand binding and inhibitory potency.

To further explore isoform-specific features relevant to ligand binding, we conducted a sequence and structural comparison between human LDHA and LDHB (Figure 7). As shown in Figure 7A, pairwise sequence alignment revealed 75.1% identity (251 out of 334 residues) and 88.6% similarity (296 out of 334 residues), with several notable substitutions in the NAD(H)- and substrate-binding domains. While the domain architecture is largely conserved, the specific residue replacements at the active site help explain the distinct kinetic behavior and inhibitor sensitivity observed between the isoforms.

In addition, a structural alignment was performed between LDHA bound to NADH (PDB ID: 4ZVV; chain A) and LDHB bound to NAD^+^ (PDB ID: 1T2F; chain A), using UCSF ChimeraX’s v1.9 MatchMaker tool (Needleman–Wunsch algorithm, BLOSUM62 matrix). The alignment produced a sequence alignment score of 1365, a pruned RMSD of 0.846 Å (over 298 atom pairs), and a global RMSD of 1.788 Å (across 331 residues), indicating strong overall structural conservation. Nevertheless, localized conformational differences were observed around the substrate-binding pocket and cofactor interface, which may contribute to isoform-specific ligand interactions (Figure 7B). The 2D interaction diagrams further show subtle differences in how NAD^+^ (LDHB) and NADH (LDHA) interact with key residues, including variations in hydrogen bonding networks and electrostatic environments. These differences are likely to influence both substrate recognition and inhibitor binding mechanisms.

Collectively, these findings highlight the importance of understanding isoform-specific structural nuances when designing or repurposing LDH inhibitors. Such insights are critical for the rational development of selective allosteric modulators targeting LDHB. It is worth mentioning, however, that while our sequence and structural alignments between LDHA and LDHB reveal critical variations at the active-site and cofactor-binding regions, these differences alone may not fully account for the isoform-specific binding preferences observed for luteolin and quercetin.

Specifically, both flavonoids act as competitive inhibitors of LDHA by binding at the active site, whereas in our study, they inhibit LDHB through uncompetitive, allosteric mechanisms. This divergence cannot be readily explained by domain-level or residue-level differences alone. Instead, it likely arises from dynamic and conformational factors—such as loop flexibility, inter-subunit communication, and allosteric site plasticity—which modulate ligand accessibility and specificity. Further structural, biochemical, and molecular dynamics studies are warranted to elucidate the mechanistic basis for this differential binding behavior. Such investigations could reveal how intrinsic isoform dynamics and ligand-induced fit contribute to selective binding, providing a rational framework for isoform-specific inhibitor design.

Another important consideration when identifying selective inhibitors of LDHB is the assay design itself. LDHB is often evaluated under the same experimental conditions as LDHA—using pyruvate as the substrate and NADH as the cofactor—despite LDHB’s physiological preference for lactate and NAD^+^. Most published LDH inhibitor studies have focused on the forward reaction (pyruvate → lactate), primarily aiming to identify inhibitors selective for LDHA, with LDHB typically included as a reference isoform.

However, efforts to identify LDHB-specific inhibitors require assay conditions that reflect the enzyme’s native catalytic direction—namely, the reverse reaction (lactate → pyruvate) in the presence of NAD^+^. In contrast, several studies continue to employ the forward reaction (pyruvate → lactate) using NADH as the cofactor. For instance, Shibata et al. [30] identified the first well-characterized uncompetitive LDHB inhibitor, AXKO-0046, using a RapidFire-MS system that monitored NADH consumption during the forward reaction. Another known LDHB inhibitor is oxamate, a pyruvate analog that acts as a competitive inhibitor by binding to the enzyme’s active site. To support such studies, our group has developed and validated a high-throughput colorimetric assay suitable for 96-well formats which enables efficient screening for selective LDHB inhibitors under physiologically relevant conditions [32].

Notably, our study has some limitations that warrant acknowledgment. First, our study adopted a targeted approach by focusing exclusively on natural compounds previously reported as LDHA or general LDH inhibitors, with the specific objective of evaluating their potential to inhibit LDHB under isoform-specific experimental conditions. This literature-guided strategy allowed for a systematic assessment of candidates with established precedent for LDH interaction, thereby providing a rational foundation for our investigations. However, this focused approach inherently constrained the chemical diversity of the screened library. By limiting our selection to compounds with documented LDH inhibitory activity, we may have overlooked numerous other natural products that could serve as promising LDHB inhibitors despite lacking prior association with LDH inhibition. The vast chemical space of natural products includes structurally diverse scaffolds and bioactive motifs that remain largely unexplored in the context of LDHB modulation. Future studies should broaden the screening scope through untargeted virtual libraries or by incorporating a wider range of phytochemicals to uncover novel scaffolds with selective LDHB activity.

Second, although molecular docking serves as a valuable tool for prioritizing potential inhibitors, exclusively relying on docking scores to filter compounds may inadvertently exclude candidates with favorable in vivo properties, including metabolic stability, membrane permeability, and bioavailability. In this study, we employed the docking score of AXKO-0046, a known LDHB inhibitor, as a reference threshold and systematically excluded compounds exhibiting lower predicted binding affinities. However, this approach may overlook compounds with modest docking scores that could nonetheless demonstrate significant biological activity through alternative binding modes not adequately captured by static docking simulations. Furthermore, the dynamic nature of protein–ligand interactions in physiological environments may differ substantially from the rigid structures used in computational docking. Therefore, future investigations should adopt a more integrative selection strategy that balances docking affinity with pharmacokinetic characteristics and chemical diversity. Such an approach may uncover additional promising LDHB inhibitors that would otherwise be dismissed based solely on computational binding predictions, ultimately enhancing the comprehensiveness and effectiveness of the screening process.

Third, although our docking and kinetic analyses support anuncompetitive allosteric inhibition mechanism for luteolin and quercetin, further investigation is required to elucidate the conformational dynamics underlying their interaction with LDHB. Future studies incorporating molecular dynamics simulations, site-directed mutagenesis, and structural analyses will be instrumental in defining these mechanisms in detail. Such insights will enable the rational optimization of these flavonoid scaffolds to enhance their inhibitory potency, isoform selectivity, and therapeutic applicability.

Finaly, while virtual screening, drug-likeness assessment, and in silico toxicity predictions provide valuable tools for prioritizing candidate compounds, these approaches have inherent limitations. For instance, camptothecin, which received the highest docking score in our virtual screening, demonstrated only modest inhibitory activity against LDHB in vitro. Furthermore, although the ProTox 3.0 and StopTox predictions suggested minimal toxicity risks, camptothecin is known to induce toxic side effects in clinical settings [62]. This discrepancy underscores the imperfect predictive power of computational models and emphasizes the critical importance of validating results through experimental assays. Additionally, compounds excluded based solely on virtual screening results may retain biological activity under specific conditions. Therefore, while our approach provides a structured and efficient strategy for candidate selection, it should be regarded as complementary—rather than definitive—within the broader drug discovery pipeline. Future work should expand experimental validation to encompass both high- and low-scoring virtual hits, particularly those exhibiting favorable safety profiles or structural novelty.

**Figure 8:**
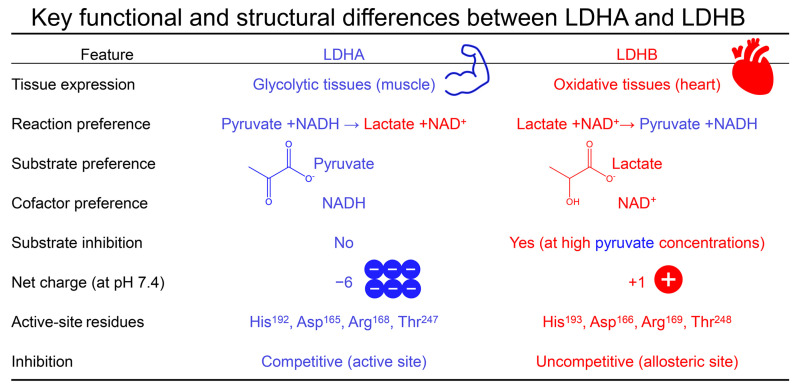
The key structural and functional differences between human LDHA and LDHB relevant to isoform-specific inhibitor design. Despite their high structural similarity, LDHA and LDHB differ in tissue distribution, substrate and cofactor preference, net charge, and inhibition mechanisms. LDHA is predominant in glycolytic tissues and catalyzes the conversion of pyruvate to lactate using NADH, while LDHB, expressed in oxidative tissues, favors the reverse reaction using NAD^+^. LDHB also shows substrate inhibition at high pyruvate levels. Importantly, LDHA is typically inhibited competitively at the active site, whereas LDHB is inhibited allosterically via uncompetitive mechanisms, as shown for luteolin and quercetin in this study. These differences highlight the need for isoform-specific assay conditions and targeted inhibitor development.

In summary, this work highlights the critical importance of isoform-specific evaluation when targeting LDH enzymes for therapeutic inhibition. Our findings further underscore the need to evaluate LDHA and LDHB under isoform-appropriate assay conditions, considering their distinct substrate preferences, tissue expression profiles, and inhibition mechanisms (Figure 8). LDHB activity is best assessed by measuring the conversion of lactate to pyruvate in the presence of NAD^+^. Although LDHA and LDHB share substantial structural and functional similarity, their differing catalytic preferences and binding-site topologies result in distinct inhibitory profiles. Our integrated in silico and in vitro approach demonstrates that flavonoids such as luteolin and quercetin—previously reported as competitive inhibitors of LDHA—act as uncompetitive inhibitors of LDHB, binding to an allosteric site at the subunit interface. These findings expand the landscape of natural compounds capable of modulating LDHB activity and emphasize the necessity of carefully optimized assay conditions to reflect isoform-specific physiology. Ultimately, our results provide a solid foundation for the rational development of phytochemical-based LDH inhibitors and may inform future efforts to design dual-target or selectively tailored therapeutics for metabolic and proliferative disorders.

## 4. Materials and Methods

### 4.1. Materials

Unless otherwise specified, all chemicals were obtained from Sigma-Aldrich (St. Louis, MO, USA). Recombinant human LDHB (Catalog #: 9205-HB) was purchased from R&D Systems (Minneapolis, MN, USA). The candidate LDHB inhibitors evaluated in vitro—including camptothecin (Catalog #: 11694), fisetin (Catalog #: 15246), luteolin (Catalog #: 10004161), and quercetin (Catalog #: 10005169)—were purchased from Cayman Chemical (Ann Arbor, MI, USA). AXKO-0046 (Catalog #: HY-147216A) was obtained from MedChemExpress (Monmouth Junction, NJ, USA). All compounds had a reported purity of ≥95%.

Stock solutions of nitro blue tetrazolium (NBT, 30 mM) and phenazine methosulfate (PMS, 30 mM) were prepared in 70% (*v*/*v*) dimethylformamide (DMF) and distilled water, respectively, and stored protected from light.

### 4.2. Preparation of Virtual Library of Natural Compounds

A virtual library of natural compounds was constructed by mining the peer-reviewed scientific literature for reported LDH inhibitors. A systematic search of the PubMed, Scopus, and Web of Science databases was conducted using combinations of keywords related to LDH, LDHA, LDHB inhibition, natural products, and compound screening. No restriction was placed on the publication date, and articles published up to February 2025 were considered. Eligible studies (Table 1) were those reporting natural or plant-derived compounds with experimentally validated LDHA and/or LDHB inhibitory activity, either through enzyme inhibition assays, molecular docking, or cell-based functional assays. Duplicates, synthetic analogs, and compounds with incomplete structural data were excluded. The curated list was used to generate a ligand dataset of 115 compounds for molecular docking against LDHB. The two-dimensional chemical structures of the phytochemicals in the Structured Data Format (SDF) were retrieved from the PubChem-NCBI database (https://pubchem.ncbi.nlm.nih.gov/, accessed on 1 April 2025) [63]. The SDF files were minimized and converted into PDB format using the OpenBabel feature of PyRx v 1.1.

### 4.3. Virtual Screening

To investigate potential LDHB inhibitors via molecular docking studies, the crystal structure of human LDHB (PDB ID: 7DBI) was retrieved from the RCSB Protein Data Bank (https://www.rcsb.org/; accessed on 2 April 2025) [30]. This structure is co-crystallized with AXKO-0046, a well-characterized uncompetitive inhibitor, and oxalate, a pyruvate analog that binds at the active site. Protein preparation was carried out using the DockPrep tool in UCSF ChimeraX v1.9 (Resource for Biocomputing, Visualization, and Informatics, University of California, San Francisco, CA, USA) [64,65], which included the addition of polar hydrogens and assignment of charges at physiological pH (7.4), as previously described [66].

Molecular docking was conducted using PyRx v1.1 [67], employing AutoDock Vina with an exhaustiveness level of 8. An initial blind docking of 115 phytochemicals previously reported as LDHA inhibitors (Supplementary Table S1) was performed across a broad region encompassing chains A and C of the LDHB tetramer. The grid box was centered at coordinates x = 16.423, y = −6.484, z = 0.176 with dimensions of 65 × 37 × 48 Å, covering both the active sites and the allosteric interface between the two subunits.

Since all test compounds localized away from the active sites and clustered near the inter-subunit interface, a second round of docking was performed using refined grid coordinates based on the binding site of AXKO-0046 (x = 15.05, y = −0.64, z = −28.79), which defines the allosteric pocket in PDB structure 7DBJ. A cubic grid box with a 22 Å side length was used for this focused docking. Compounds exhibiting binding affinities better than or equal to −7.6 kcal/mol—the docking score of AXKO-0046—were selected for further analysis.

For comparative analysis, molecular docking was also performed against the active site of human LDHA using the crystal structure with PDB ID 4ZVV (chain A), which is co-crystallized with the potent LDHA inhibitor (R)-GNE-140 (PubChem CID: 121225870) [53]. The docking grid was centered at x = 42.02, y = 14.07, z = 25.18, corresponding to the active site of GNE-140 as described by Shu et al. [55]. The grid box dimensions were set to 27.36 Å per side to ensure the full coverage of the active site [55].

All docking results were analyzed and visualized using BIOVIA Discovery Studio Visualizer 2024 (Dassault Systèmes, San Diego, CA, USA).

### 4.4. ADME and Toxicity Assessment of Selected Phytochemicals

Following the initial virtual screening, 16 phytochemicals were selected for the further evaluation of their drug-like properties through ADME/T (absorption, distribution, metabolism, excretion, and toxicity) profiling. Pharmacokinetic parameters were predicted using the SwissADME web server (http://www.swissadme.ch/; accessed on 3 April 2025), which assesses critical descriptors such as lipophilicity, solubility, gastrointestinal absorption, and blood–brain barrier permeability. Canonical SMILES notations for each compound were retrieved from PubChem-NCBI (https://pubchem.ncbi.nlm.nih.gov/, accessed on 3 April 2025) and input into SwissADME for property prediction. Toxicological profiles were evaluated using two complementary platforms, ProTox 3.0 (https://tox.charite.de/protox3/index.php?site=home#) [68] and StopTox (https://stoptox.mml.unc.edu/) [69], both accessed on 5 April 2025, to ensure the reliability and consistency of the results.

ProTox 3.0 employs a combination of machine learning algorithms, molecular similarity assessments, fragment propensity analysis, and structural alerts to predict toxicity endpoints including acute toxicity, hepatotoxicity, carcinogenicity, mutagenicity, immunotoxicity, cytotoxicity, and Tox21 pathway interference. StopTox utilizes a suite of validated QSAR models based on curated datasets to predict adverse effects such as acute oral and inhalation toxicity, skin sensitization, and eye irritation or corrosion. Together, these tools provided a comprehensive preclinical safety and pharmacokinetic assessment of the selected lead compounds.

### 4.5. LDHB Activity Assay

To assess the inhibitory potential of the best compounds, namely camptothecin, fisetin, luteolin, and quercetin, identified through virtual screening and ADME/T profiling, a previously validated colorimetric endpoint assay developed by our group was employed in a 96-well plate format [31,32]. This assay quantifies LDHB activity by measuring NADH generation resulting from the conversion of lactate to pyruvate, a reaction catalyzed by LDHB. The produced NADH reacts with nitro blue tetrazolium (NBT) in the presence of phenazine methosulfate (PMS), generating a blue-purple formazan dye detectable at approximately 570 nm.

The reaction was performed in a buffer containing 50 mM CHES (pH 9.6), 150 mM NaCl, 300 μM NBT, 30 μM PMS, and 0.13% gelatin (used to prevent formazan precipitation). Each well was loaded with 90 μL of the reaction buffer followed by 25 μL of a substrate solution comprising 10 mM NAD^+^ and 250 mM sodium lactate in the same buffer. Next, 5 μL of the test compound at varying concentrations was added; the buffer alone was used in the control wells. The plates were incubated for 30 s at 25 °C in the dark before measuring the initial absorbance at 570 nm (A_initial_) using a microplate reader (PerkinElmer, Waltham, MA, USA). The reaction was initiated by adding 2 μL of 7.5 μM LDHB. After a 5 min incubation with continuous shaking, a second absorbance measurement (A_final_) was taken.

The difference in absorbance (ΔA_570_ = A_final_ − A_initial_) was used to calculate the amount of NADH produced by referencing a standard curve (0–15 nmol NADH). Readings were corrected for background using a blank lacking LDHB. One unit of enzyme activity was defined as the amount of enzyme producing 1 μmol of NADH per minute at 25 °C. LDHB activity (mU/mL) was calculated using the following Equation (1):

(1) $$LDH\;activity(\frac{mU}{mL})=\frac{\left[NADH](nmol\right)}{\left(T_{final-}T_{initial}\right)(min)\times V(mL)}=\frac{nmol}{\min\times mL}$$

where [NADH] is the amount (in nmol) of NADH that is released between T_final_ and T_initial_, and V is the sample volume (LDHB in mL) added in each well of the 96-well plate.

IC_50_ values for each compound were calculated using GraphPad Prism v8.0.2 (GraphPad Software Inc., San Diego, CA, USA) by fitting dose–response curves to the enzymatic activity data via nonlinear regression analysis. All the measurements were performed in triplicate.

To determine the mode of LDHB inhibition by luteolin and quercetin—the two most potent inhibitors identified in this study—we conducted detailed enzymatic assays using varying concentrations of substrate (lactate) or cofactor (NAD^+^). These kinetic studies followed the classical Michaelis–Menten framework. For NAD^+^ titration, assays were performed using eight NAD^+^ concentrations (120, 240, 360, 480, 700, 900, 1000, and 1200 μM) in the presence of 25 mM sodium lactate. For lactate titration, eight lactate concentrations (2.5, 5, 10, 15, 20, 25, 37.5, and 50 mM) were evaluated with a fixed NAD^+^ concentration of 1 mM. Each reaction was carried out with 2.5 nM LDHB and increasing concentrations of either inhibitor (5–100 μM), followed by incubation at room temperature for 6.5 min. Initial reaction velocities were determined and fitted to a nonlinear Michaelis–Menten model to extract apparent kinetic parameters (K_m_ and V_max_) using GraphPad Prism v8.0.2 (GraphPad Software Inc., San Diego, CA, USA). Lineweaver–Burk plots (1/V vs. 1/[S]) were also generated to visualize shifts in enzyme kinetics. To complement these analyses, the data were further examined using a mixed-model inhibition approach within GraphPad Prism. This model, which avoids the distortions associated with reciprocal transformations, evaluates inhibition kinetics using polynomial regression and is governed by the following equations [70]. The mixed-model fit is described by Equations (2)–(4), and contains competitive, uncompetitive, and noncompetitive inhibition terms:

(2) $$V_{max}^{app}=V_{max}/(1+\frac{I}{a\cdot K_{i}})$$

(3) $$K_{m}^{app}=K_{m}\cdot (1+\frac{I}{K_{i}})/(1+\frac{I}{a\cdot K_{i}})$$

(4) $$Y=V_{max}^{app}\cdot X/(K_{m}^{app}+X)$$

In the above equations, $V_{max}^{app}$ and $K_{m}^{app}$ are the maximum enzyme activity and Michaelis–Menten constant, respectively, in the presence of an inhibitor; V_max_ and K_m_ represent the maximum enzyme velocity and Michaelis–Menten constant, respectively, in the absence of an inhibitor; and K*_i_* is the inhibition constant. In addition, X is the substrate concentration, Y is the enzyme activity, I is the inhibitor concentration, and alpha (α) is a coefficient that defines the mechanism of inhibition. An α value of 1 indicates noncompetitive inhibition; α ≫ 1 suggests a competitive mechanism; and α < 1 (but >0) indicates uncompetitive inhibition.

### 4.6. Statistical Analysis

Unless otherwise stated, the experiments were carried out in triplicate, and the data are presented as mean values ± standard deviation (SD). Statistical analysis was performed using one-way ANOVA, followed by Dunnett’s test for multiple comparisons (Supplementary Figures S1 and S2). Statistical significance was set at *p* < 0.05. The analysis was conducted using GraphPad Prism v.8.0.2 (GraphPad Software Inc., San Diego, CA, USA).

## 5. Conclusions

This study identifies the natural flavonoids luteolin and quercetin as uncompetitive allosteric inhibitors of human LDHB, thereby expanding current understandings of isoform-specific LDH regulation. Although both compounds have previously been characterized as competitive inhibitors of LDHA, our integrated in silico and in vitro analyses reveal that they exhibit distinct inhibitory behavior toward LDHB by binding to an allosteric site located at the dimer interface. These findings underscore the structural and functional differences between LDHA and LDHB, emphasizing the importance of isoform-specific assay conditions, particularly the use of lactate and NAD^+^ for LDHB activity measurements. Furthermore, the implementation of a validated high-throughput colorimetric assay enables the efficient screening of LDHB-selective inhibitors under physiologically relevant conditions. Collectively, this work not only highlights the potential of repurposing natural compounds for LDHB inhibition but also provides a foundation for the rational design of isoform-selective therapeutics targeting metabolic dysregulation in cancer and other diseases.

## Figures and Tables

**Table 1:** Studies examining natural products as potential LDH inhibitors with diverse structural and pharmacological characteristics.

Study	Main Outcomes	Reference
1	A review on the potential of natural compounds as LDH inhibitors.	[38]
2	A screening of LDH inhibitors from natural bioactive compounds using electrophoretically mediated microanalysis (EMMA). Quercetin, luteolin, and ursolic acid were identified as potent LDH inhibitors.	[50]
3	A review of LDH inhibition potential across diverse structural classes including phenolics, alkaloids, and carotenoids.	[39]
4	A screening of potential LDHA inhibitors from *Polygala tenuifolia*. Sibiricose A5, 3,6′-di-*O*-sinapoyl-sucrose, glomeratose A, tenuifoliside B, and tenuifoliside C emerged as LDHA inhibitors	[51]
5	The inhibitory effects of 17 flavonoids on LDHA were analyzed, with fisetin demonstrating the strongest activity.	[44]
6	A magnetic nanoparticle-based assay was employed to identify LDH inhibitors from *Rhubarb* and *Polygonum cuspidatum*. In total, 12 potential inhibitors were successfully detected and characterized.	[52]
7	A total of 52 phytochemicals derived from the plant species *Oroxylum indicum* (L.) Kurz were screened against LDHA using a bioinformatics approach. Oroxindin, Chrysin-7-*O*-glucuronide, and Oroxin A were identified as potential inhibitors.	[45]

**Table 2:** Predicted physicochemical and ADME properties of top four candidate compounds selected for LDHB inhibition.

Parameters		Camptothecin	Fisetin	Luteolin	Quercetin
Physicochemical Properties	Formula	C_20_H_16_N_2_O_4_	C_15_H_10_O_6_	C_15_H_10_O_6_	C_15_H_10_O_7_
	Molecular weight	348.35	286.24	286.24	302.24
	H-bond acceptors	5	6	6	7
	H-bond donors	1	1	4	5
	Molar refractivity	95.31	76.01	76.01	78.03
	TPSA (A)	81.42	111.13	111.13	131.36
Lipophilicity	Log P_o/w_ (iLOGP)	2.49	1.5	1.86	1.63
	Log P_o/w_ (XLOGP3)	1.74	1.97	2.53	1.54
	Log P_o/w_ (WLOGP)	1.82	2.28	2.28	1.99
	Log P_o/w_ (MLOGP)	1.64	−0.03	−0.03	−0.56
	Log P_o/w_ (SILICOS-IT)	3.29	2.03	2.03	1.54
	Consensus Log P_o/w_	2.2	1.55	1.73	1.23
Water Solubility	Log S (SILICOS-IT)	−5.83	−3.35	−3.82	−3.82
	Solubility (mg/mg; mol/L)	5.20 × 10^−4^; 1.49 × 10^−6^	1.27 × 10^−1^; 4.43 × 10^−4^	4.29 × 10^−2^; 1.50 × 10^−4^	1.73 × 10^−1^; 5.73 × 10^−4^
	Class	Moderately soluble	Soluble	Soluble	Soluble
Pharmacokinetics	GI absorption	High	High	High	High
	BBB permeant	No	No	No	No
	P-gp substrate	Yes	No	No	No
	CYP1A2 inhibitor	Yes	Yes	Yes	Yes
	CYP2C19 inhibitor	No	No	No	No
	CYP2C9 inhibitor	Yes	No	No	No
	CYP2D6 inhibitor	No	Yes	Yes	Yes
	CYP3A4 inhibitor	Yes	Yes	Yes	Yes
	Log Kp (skin permeation) (cm/s)	−7.19	−6.65	−6.25	−7.05
Drug-Likeness	Lipinski	Yes; 0 violations	Yes; 0 violations	Yes; 0 violations	Yes; 0 violations
	Ghose	Yes	Yes	Yes	Yes
	Bioavailability score	0.55	0.55	0.55	0.55
Medicinal Chemistry	PAINS	0 alerts	1 alert: catechol_A	1 alert: catechol_A	1 alert: catechol_A
	Lead-likeness	Yes	Yes	Yes	Yes
	Synthetic accessibility	3.84	3.16	3.16	3.23

**Table 3:** In silico toxicity prediction of selected LDHB inhibitors using ProTox 3.0 ^1^.

Classification	Target	Camptothecin	Fisetin	Luteolin	Quercetin
Organ toxicity	Hepatotoxicity	Inactive (0.83)	Inactive (0.70)	Inactive (0.69)	Inactive (0.69)
	Neurotoxicity	Active (0.74)	Inactive (0.88)	Inactive (0.89)	Inactive (0.89)
	Nephrotoxicity	Active (0.61)	Active (0.57)	Active (0.62)	Active (0.62)
	Respiratory toxicity	Active (0.70)	Active (0.82)	Active (0.83)	Active (0.83)
	Cardiotoxicity	Inactive (0.73)	Inactive (0.93)	Inactive (0.99)	Inactive (0.99)
Toxicity endpoints	Carcinogenicity	Inactive (0.57)	Active (0.71)	Active (0.68)	Active (0.68)
	Immunotoxicity	Active (0.99)	Inactive (0.51)	Inactive (0.97)	Inactive (0.87)
	Mutagenicity	Inactive (0.59)	Inactive (0.53)	Active (0.51)	Active (0.51)
	Cytotoxicity	Active (0.98)	Inactive (0.98)	Inactive (0.99)	Inactive (0.99)
	Ecotoxicity	Inactive (0.63)	Inactive (0.54)	Inactive (0.53)	Inactive (0.53)
	Clinical toxicity	Active (0.77)	Inactive (0.54)	Inactive (0.53)	Inactive (0.53)
Tox21–nuclear receptor signaling pathways	Aryl hydrocarbon Receptor (AhR)	Inactive (0.91)	Active (0.84)	Active (0.91)	Active (0.91)
	Androgen receptor	Inactive (0.99)	Inactive (0.99)	Inactive (0.99)	Inactive (0.99)
	Androgen receptor ligand-binding domain (AR-LBD)	Inactive (0.98)	Inactive (0.72)	Inactive (0.97)	Inactive (0.97)
	Aromatase	Inactive (0.66)	Inactive (0.88)	Inactive (0.91)	Inactive (0.91)
	Estrogen receptor alpha	Inactive (0.95)	Active (0.69)	Active (0.87)	Active (0.87)
	Estrogen receptor ligand-binding domain (ER-LBD)	Inactive (0.99)	Active (0.86)	Active (0.95)	Active (0.95)
	Peroxisome proliferator activated receptor gamma	Inactive (0.93)	Inactive (0.98)	Inactive (0.98)	Inactive (0.98)
Tox21–stress response pathways	Nuclear factor (erythroid-derived 2)-like 2/antioxidant responsive element	Inactive (0.69)	Inactive (0.98)	Inactive (0.99)	Inactive (0.99)
	Heat shock factor response element	Inactive (0.69)	Inactive (0.98)	Inactive (0.99)	Inactive (0.99)
	Mitochondrial membrane potential	Inactive (0.58)	Active (0.82)	Active (1.00)	Active (1.00)
	Phosphoprotein (Tumor Suppressor) p53	Active (0.54)	Inactive (0.97)	Inactive (0.97)	Inactive (0.97)
	ATPase family AAA domain-containing protein 5 (ATAD5)	Inactive (0.98)	Inactive (0.77)	Inactive (0.99)	Inactive (0.99)

^1^ Predicted toxicity outcomes from ProTox 3.0 shown as Active or Inactive, with confidence scores (probability) in parentheses.

**Table 4:** Systemic and topical toxicity prediction of selected LDHB inhibitors using StopTox server ^1^.

Endpoint	Camptothecin	Fisetin	Luteolin	Quercetin
Acute inhalation toxicity	Non-toxic (74%)	Non-Toxic (67%)	Non-Toxic (69%)	Non-Toxic (69%)
Acute oral toxicity	Toxic (90%)	Non-Toxic (75%)	Non-Toxic (78%)	Non-Toxic (72%)
Acute dermal toxicity	Non-Toxic (68%)	Toxic (69%)	Toxic (64%)	Toxic (64%)
Eye irritation and corrosion	Toxic (71%)	Toxic (57%)	Toxic (51%)	Toxic (51%)
Skin sensitization	Non-Sensitizer (70%)	Sensitizer (80%)	Sensitizer (70%)	Sensitizer (70%)
Skin irritation and corrosion	Negative (70%)	Negative (90%)	Negative (80%)	Negative (80%)

^1^ Toxicity classifications are based on StopTox predictions, with confidence scores (%) shown in parentheses.

**Table 5:** Effect of luteolin and quercetin on K_m_ and V_max_ values of lactate and NAD^+^ in LDHB activity ^1^.

	Luteolin (μΜ)							
	Control	10	20	30	40	50	75	100
Lactate								
V_max_ (mU/mL)	1020 ± 28	942 ± 31	888 ± 16	832 ± 21	749.2 ± 17	624 ± 24	506 ± 15	406 ± 22
K_m_ (mΜ)	18.6 ± 1.6	17.6 ± 1.3	17.2 ± 1.6	16.5 ± 1.2	15.9 ± 1.1	14.3 ± 2.1	10.5 ± 0.9	8.5 ± 1.0
NAD^+^								
V_max_ (mU/mL)	1006 ± 35	945 ± 27	868 ± 35	774 ± 32	664 ± 40	563 ± 29	456 ± 27	417 ± 22
K_m_ (μΜ)	311 ± 19	309 ± 19	299 ± 21	286 ± 25	258 ± 32	238 ± 22	189 ± 19	173 ± 15
	**Quercetin (μΜ)**							
	**Control**	**10**	**20**	**30**	**40**	**50**	**75**	**100**
Lactate								
V_max_ (mU/mL)	1020 ± 28	938 ± 29	881 ± 19	791 ± 37	689 ± 53	621 ± 23	557 ± 37	478 ± 26
K_m_ (mΜ)	18.6 ± 1.6	17.1 ± 2.0	16.5 ± 1.9	15.8 ± 1.4	14.4 ± 1.1	13.3 ± 1.3	12.2 ± 1.1	11.8 ± 1.3
NAD^+^								
V_max_ (mU/mL)	1006 ± 35	952 ± 36	861 ± 22	761 ± 52	701 ± 44	605 ± 46	504 ± 44	454 ± 33
K_m_ (μΜ)	311 ± 19	298 ± 29	267 ± 24	254 ± 27	220 ± 19	209 ± 25	178 ± 16	158 ± 15

^1^ Results expressed as mean ± SD from three independent experiments.

**Table 6:** LDHB inhibition data obtained with luteolin and quercetin with lactate and NAD^+^.

Parameter	Luteolin		Quercetin	
	Lactate	NAD^+^	Lactate	NAD^+^
Κi (μM)	349	194	265	539
α ^a^	0.21	0.34	0.31	0.14
r^2 b^	0.9868	0.9802	0.9908	0.9857

^a^ The value of the alpha (α) factor is indicative of the mechanism of inhibition, as described in the text. ^b^ Goodness of fit.

## Data Availability

The data that support the findings of this study are available from the corresponding author [C.P.] on special request.

## Supplementary Materials

The following supporting information can be downloaded at: https://www.mdpi.com/article/10.3390/molecules30142923/s1, Table S1: The 115 natural compounds identified as potential LDHA inhibitors and screened against LDHB; Figure S1: The assessment of the four most promising LDHB inhibitor candidates, identified through in silico analysis and drug-likeness evaluations, on the formation of blue-purple formazan; Figure S2: The effect of pre-incubation time on the inhibitory activity of luteolin (A) and quercetin (B) on LDHB activity.

## References

[1] Farhana A., Lappin S.L. (2022). Biochemistry, lactate dehydrogenase. StatPearls.

[2] Gladden L.B. (2004). Lactate metabolism: A new paradigm for the third millennium. J. Physiol..

[3] Adeva M., González-Lucán M., Seco M., Donapetry C. (2013). Enzymes involved in l-lactate metabolism in humans. Mitochondrion.

[4] Maeda M., Ko M., Mane M.M., Cohen I.J., Shindo M., Vemuri K., Serganova I., Blasberg R. (2022). Genetic and drug inhibition of LDH-A: Effects on murine gliomas. Cancers.

[5] Read J.A., Winter V.J., Eszes C.M., Sessions R.B., Brady R.L. (2001). Structural basis for altered activity of M- and H-isozyme forms of human lactate dehydrogenase. Proteins.

[6] Papaneophytou C. (2024). The Warburg effect: Is it always an enemy?. Front. Biosci..

[7] Hanahan D., Weinberg R.A. (2011). Hallmarks of cancer: The next generation. Cell.

[8] Liu J., Zhang C., Zhang T., Chang C.Y., Wang J., Bazile L., Zhang L., Haffty B.G., Hu W., Feng Z. (2022). Metabolic enzyme LDHA activates Rac1 GTPase as a noncanonical mechanism to promote cancer. Nat. Metab..

[9] Sharma D., Singh M., Rani R. (2022). Role of LDH in tumor glycolysis: Regulation of LDHA by small molecules for cancer therapeutics. Semin. Cancer Biol..

[10] Ždralević M., Marchiq I., de Padua M.M.C., Parks S.K., Pouysségur J. (2017). Metabolic plasiticy in cancers-distinct role of glycolytic enzymes GPI, LDHs or membrane transporters MCTs. Front. Oncol..

[11] Claps G., Faouzi S., Quidville V., Chehade F., Shen S., Vagner S., Robert C. (2022). The multiple roles of LDH in cancer. Nat. Rev. Clin. Oncol..

[12] Dennison J.B., Molina J.R., Mitra S., González-Angulo A.M., Balko J.M., Kuba M.G., Sanders M.E., Pinto J.A., Gómez H.L., Arteaga C.L. (2013). Lactate dehydrogenase B: A metabolic marker of response to neoadjuvant chemotherapy in breast cancer. Clin. Cancer Res..

[13] McCleland M.L., Adler A.S., Shang Y., Hunsaker T., Truong T., Peterson D., Torres E., Li L., Haley B., Stephan J.-P. (2012). An integrated genomic screen identifies LDHB as an essential gene for triple-negative breast cancer. Cancer Res..

[14] McCleland M.L., Adler A.S., Deming L., Cosino E., Lee L., Blackwood E.M., Solon M., Tao J., Li L., Shames D. (2013). Lactate dehydrogenase B is required for the growth of KRAS-dependent lung adenocarcinomas. Clin. Cancer Res..

[15] Li C., Chen Y., Bai P., Wang J., Liu Z., Wang T., Cai Q. (2016). LDHB may be a significant predictor of poor prognosis in osteosarcoma. Am. J. Transl. Res..

[16] Zha X., Wang F., Wang Y., He S., Jing Y., Wu X., Zhang H. (2011). Lactate dehydrogenase B is critical for hyperactive mTOR-mediated tumorigenesis. Cancer Res..

[17] Xu X., Pan X., Fan Z., Xia J., Ren X. (2024). Lactate dehydrogenase B as a metabolism-related marker for immunotherapy in head and neck squamous cell carcinoma. Cell. Signal..

[18] Hu X., Huang Z., Li L. (2025). LDHB mediates histone lactylation to activate PD-L1 and promote ovarian cancer immune escape. Cancer Investig..

[19] Brisson L., Bański P., Sboarina M., Dethier C., Danhier P., Fontenille M.J., Van Hée V.F., Vazeille T., Tardy M., Falces J. (2016). Lactate dehydrogenase B controls lysosome activity and autophagy in cancer. Cancer Cell.

[20] Sui X., Chen R., Wang Z., Huang Z., Kong N., Zhang M., Han W., Lou F., Yang J., Zhang Q. (2013). Autophagy and chemotherapy resistance: A promising therapeutic target for cancer treatment. Cell Death Dis..

[21] Li Y.J., Lei Y.H., Yao N., Wang C.R., Hu N., Ye W.C., Zhang D.M., Chen Z.S. (2017). Autophagy and multidrug resistance in cancer. Chin. J. Cancer.

[22] Shi L., Yan H., An S., Shen M., Jia W., Zhang R., Zhao L., Huang G., Liu J. (2019). SIRT5-mediated deacetylation of LDHB promotes autophagy and tumorigenesis in colorectal cancer. Mol. Oncol..

[23] Sonveaux P., Végran F., Schroeder T., Wergin M.C., Verrax J., Rabbani Z.N., De Saedeleer C.J., Kennedy K.M., Diepart C., Jordan B.F. (2008). Targeting lactate-fueled respiration selectively kills hypoxic tumor cells in mice. J. Clin. Investig..

[24] Kennedy K.M., Scarbrough P.M., Ribeiro A., Richardson R., Yuan H., Sonveaux P., Landon C.D., Chi J.T., Pizzo S., Schroeder T. (2013). Catabolism of exogenous lactate reveals it as a legitimate metabolic substrate in breast cancer. PLoS ONE.

[25] Dhup S., Dadhich R.K., Porporato P.E., Sonveaux P. (2012). Multiple biological activities of lactic acid in cancer: Influences on tumor growth, angiogenesis and metastasis. Curr. Pharm. Des..

[26] Leite T.C., Coelho R.G., Da Silva D., Coelho W.S., Marinho-Carvalho M.M., Sola-Penna M. (2011). Lactate downregulates the glycolytic enzymes hexokinase and phosphofructokinase in diverse tissues from mice. FEBS Lett..

[27] Altinoz M.A., Ozpinar A. (2022). Oxamate targeting aggressive cancers with special emphasis to brain tumors. Biomed. Pharmacother..

[28] Li X., Lu W., Hu Y., Wen S., Qian C., Wu W., Huang P. (2013). Effective inhibition of nasopharyngeal carcinoma in vitro and in vivo by targeting glycolysis with oxamate. Int. J. Oncol..

[29] Cheng A., Zhang P., Wang B., Yang D., Duan X., Jiang Y., Xu T., Jiang Y., Shi J., Ding C. (2019). Aurora-A mediated phosphorylation of LDHB promotes glycolysis and tumor progression by relieving the substrate-inhibition effect. Nat. Commun..

[30] Shibata S., Sogabe S., Miwa M., Fujimoto T., Takakura N., Naotsuka A., Kitamura S., Kawamoto T., Soga T. (2021). Identification of the first highly selective inhibitor of human lactate dehydrogenase B. Sci. Rep..

[31] Vlasiou M., Nicolaidou V., Papaneophytou C. (2023). Targeting lactate dehydrogenase-B as a strategy to fight cancer: Identification of potential inhibitors by In silico analysis and in vitro screening. Pharmaceutics.

[32] Papaneophytou C., Zervou M.-E., Theofanous A. (2021). Optimization of a colorimetric assay to determine lactate dehydrogenase B activity using design of experiments. SLAS Discov..

[33] Nasim N., Sandeep I.S., Mohanty S. (2022). Plant-derived natural products for drug discovery: Current approaches and prospects. Nucleus.

[34] Seidel V. (2020). Plant-derived chemicals: A source of inspiration for new drugs. Plants.

[35] Hua F., Shang S., Hu Z.W. (2017). Seeking new anti-cancer agents from autophagy-regulating natural products. J. Asian Nat. Prod. Res..

[36] Lee D.Y., Song M.Y., Kim E.H. (2021). Role of oxidative stress and Nrf2/KEAP1 signaling in colorectal cancer: Mechanisms and therapeutic perspectives with phytochemicals. Antioxidants.

[37] Feng Y., Xiong Y., Qiao T., Li X., Jia L., Han Y. (2018). Lactate dehydrogenase A: A key player in carcinogenesis and potential target in cancer therapy. Cancer Med..

[38] Han J.H., Lee E.J., Park W., Ha K.T., Chung H.S. (2023). Natural compounds as lactate dehydrogenase inhibitors: Potential therapeutics for lactate dehydrogenase inhibitors-related diseases. Front. Pharmacol..

[39] Yao H., Yang F., Li Y. (2022). Natural products targeting human lactate dehydrogenases for cancer therapy: A mini review. Front. Chem..

[40] Ikeda M. (1990). Inhibition kinetics of NAD-linked enzymes by gossypol acetic acid. Andrologia.

[41] Gomez M.S., Piper R.C., Hunsaker L.A., Royer R.E., Deck L.M., Makler M.T., Vander Jagt D.L. (1997). Substrate and cofactor specificity and selective inhibition of lactate dehydrogenase from the malarial parasite *P. falciparum*. Mol. Biochem. Parasit.

[42] Yu Y., Deck J.A., Hunsaker L.A., Deck L.M., Royer R.E., Goldberg E., Vander Jagt D.L. (2001). Selective active site inhibitors of human lactate dehydrogenases A4, B4, and C4. Biochem. Pharmacol..

[43] Di Magno L., Coluccia A., Bufano M., Ripa S., La Regina G., Nalli M., Di Pastena F., Canettieri G., Silvestri R., Frati L. (2022). Discovery of novel human lactate dehydrogenase inhibitors: Structure-based virtual screening studies and biological assessment. Eur. J. Med. Chem..

[44] Yırtıcı Ü. (2024). Natural flavonoids as promising lactate dehydrogenase A inhibitors: Comprehensive in vitro and in silico analysis. Arch. Pharm..

[45] Ahmed S.S., Rahman M.O., Alqahtani A.S., Sultana N., Almarfadi O.M., Ali M.A., Lee J. (2023). Anticancer potential of phytochemicals from *Oroxylum indicum* targeting Lactate Dehydrogenase A through bioinformatic approach. Toxicol. Rep..

[46] Granchi C., Bertini S., Macchia M., Minutolo F. (2010). Inhibitors of lactate dehydrogenase isoforms and their therapeutic potentials. Curr. Med. Chem..

[47] McInnes C. (2007). Virtual screening strategies in drug discovery. Curr. Opin. Chem. Biol..

[48] Truchon J.F., Bayly C.I. (2007). Evaluating virtual screening methods: Good and bad metrics for the “early recognition” problem. J. Chem. Inf. Model..

[49] Sadybekov A.V., Katritch V. (2023). Computational approaches streamlining drug discovery. Nature.

[50] Li W., Cui X., Chen Z. (2021). Screening of lactate dehydrogenase inhibitor from bioactive compounds in natural products by electrophoretically mediated microanalysis. J. Chromatogr. A.

[51] Li S., Liu S., Liu Z., Liu C., Song F., Pi Z. (2017). Bioactivity screening, extraction, and separation of lactate dehydrogenase inhibitors from *Polygala tenuifolia* Willd. based on a hyphenated strategy. J. Sep. Sci..

[52] Cheng G., Pi Z., Zheng Z., Liu S., Liu Z., Song F. (2020). Magnetic nanoparticles-based lactate dehydrogenase microreactor as a drug discovery tool for rapid screening inhibitors from natural products. Talanta.

[53] Boudreau A., Purkey H.E., Hitz A., Robarge K., Peterson D., Labadie S., Kwong M., Hong R., Gao M., Del Nagro C. (2016). Metabolic plasticity underpins innate and acquired resistance to LDHA inhibition. Nat. Chem. Biol..

[54] Friberg A., Rehwinkel H., Nguyen D., Pütter V., Quanz M., Weiske J., Eberspächer U., Heisler I., Langer G. (2020). Structural evidence for isoform-selective allosteric inhibition of lactate dehydrogenase A. ACS Omega.

[55] Shu Y., Yue J., Li Y., Yin Y., Wang J., Li T., He X., Liang S., Zhang G., Liu Z. (2024). Development of human lactate dehydrogenase a inhibitors: High-throughput screening, molecular dynamics simulation and enzyme activity assay. J. Comput. Aided Mol. Des..

[56] Vesell E.S. (1965). Lactate dehydrogenase Isozymes: Substrate inhibition in various human tissues. Science.

[57] Vanderlinde R.E. (1985). Measurement of total lactate dehydrogenase activity. Ann. Clin. Lab. Sci..

[58] Ždralević M., Brand A., Di Ianni L., Dettmer K., Reinders J., Singer K., Peter K., Schnell A., Bruss C., Decking S.M. (2018). Double genetic disruption of lactate dehydrogenases A and B is required to ablate the “Warburg effect” restricting tumor growth to oxidative metabolism. J. Biol. Chem..

[59] Urbańska K., Orzechowski A. (2019). Unappreciated role of LDHA and LDHB to control apoptosis and autophagy in tumor cells. Int. J. Mol. Sci..

[60] Osis G., Traylor A.M., Black L.M., Spangler D., George J.F., Zarjou A., Verlander J.W., Agarwal A. (2021). Expression of lactate dehydrogenase A and B isoforms in the mouse kidney. Am. J. Physiol. Renal Physiol..

[61] Świderek K., Paneth P. (2011). Differences and similarities in binding of pyruvate and l-lactate in the active site of M4 and H4 isoforms of human lactate dehydrogenase. Arch. Biochem. Biophys..

[62] Wang X., Zhuang Y., Wang Y., Jiang M., Yao L. (2023). The recent developments of camptothecin and its derivatives as potential anti-tumor agents. Eur. J. Med. Chem..

[63] Sayers E.W., Barrett T., Benson D.A., Bolton E., Bryant S.H., Canese K., Chetvernin V., Church D.M., DiCuccio M., Federhen S. (2010). Database resources of the National Center for Biotechnology Information. Nucleic Acids Res..

[64] Pettersen E.F., Goddard T.D., Huang C.C., Couch G.S., Greenblatt D.M., Meng E.C., Ferrin T.E. (2004). UCSF Chimera-a visualization system for exploratory research and analysis. J. Comput. Chem..

[65] Pettersen E.F., Goddard T.D., Huang C.C., Meng E.C., Couch G.S., Croll T.I., Morris J.H., Ferrin T.E. (2021). UCSF ChimeraX: Structure visualization for researchers, educators, and developers. Protein Sci..

[66] Theerawatanasirikul S., Lekcharoensuk P., Roy K. (2021). Virtual screening of catural compounds targeting proteases of coronaviruses and picornaviruses. Silico Modeling of Drugs Against Coronaviruses: Computational Tools and Protocols.

[67] Dallakyan S., Olson A.J., Hempel J.E., Williams C.H., Hong C.C. (2015). Small-molecule library screening by docking with PyRx. Chemical Biology: Methods and Protocols.

[68] Banerjee P., Kemmler E., Dunkel M., Preissner R. (2024). ProTox 3.0: A webserver for the prediction of toxicity of chemicals. Nucleic Acids Res..

[69] Borba J.V.B., Alves V.M., Braga R.C., Korn D.R., Overdahl K., Silva A.C., Hall S.U.S., Overdahl E., Kleinstreuer N., Strickland J. (2022). STopTox: An in silico alternative to animal testing for acute systemic and topical toxicity. Environ. Health Perspect..

[70] Copeland R.A. (2000). Reversible inhibitors. Enzymes: A Practical Introduction to Structure, Mechanism, and Data Analysis.

