# Supplementary material for: Flipping the Target: Evaluating Natural LDHA Inhibitors for Selective LDHB Modulation
Source: Molecules. 2025 Jul 10;30(14):2923. doi: 10.3390/molecules30142923 (PMC12299506; doi:10.3390/molecules30142923)
Supplement: Supplementary file 1 [file molecules-30-02923-s001.zip › molecules-3730864-supplementary.pdf]

Supplementary Data

Supplementary Table S1. The 115 natural compounds identified as potential LDHA inhibitors, and screened against LDHB

	Compound	PubChem CID
1	(-)-Epigallocatechin	72277
2	(-)-Epigallocatechin gallate (EGCG)	65064
3	(+)-catechin	9064
4	2-Isopropenyl-2,3-dihydronaphtho[2,3-b]furan-4,9-dione (Dehydroiso-alpha-lapachone)	364109
5	2-Methyl-6-(4-methylphenyl)hept-2-en-4-one	558221
6	3,4-Dihydroxybenzoic acid	72
7	3,6′-Di-O-sinapoylsucrose	166642520
8	4-Hydroxy-2-nonenal	5283344
9	4-Hydroxybenzoic acid	135
10	5-Hydroxy-7-methoxy-2-phenylchroman-4-one	4101463
11	6''-O-Acetyldaidzin	156155
12	6''-O-Acetylglycitin	10228095
13	6''-O-Malonylgenistin	15934091
14	Adenosine	60961
15	Aloe-emodin	10207
16	Anthraquinone	6780
17	Apigenin	5280443
18	Apigenin 7-apioglucoside (Apiin)	5280746
19	Apigenin 7-O-β-D-glucoside	12304093
20	Baicalein	5281605
21	Baicalein 6-O-glucoside	5321896
22	Baicalein 7-O-diglucoside (Oroxin B)	10077207
23	Baicalein-7-O-glucoside (Oroxin A)	5320313
24	Baicalin	64982
25	Balanophonin	23252258
26	Berberine	2353
27	Beta-Hydroxypropiovanillone	75142
28	Beta-sitosterol	222284
29	Betulin	72326
30	Betulinic acid	64971
31	Biochanin A	5280373
32	Camptothecin	24360
33	Chrysin	5281607
34	Chrysin-7-O-glucuronide	14135335

35	Crocetin	5281232
36	Curcumin	969516
37	Daidzein	5281708
38	Daidzin	107971
39	Dehydropachymic acid	15226717
40	Dehydrotrametenolic acid	15250826
41	Dihydrobaicalein	9816931
42	Dihydrooroxylin A	177032
43	Dihydropinosylvin	442700
44	Diosmetin	5281612
45	Eburicoic acid	73402
46	Echinulin	115252
47	Ellagic acid	5281855
48	Epicatechin	72276
49	Epigallocatechin	72277
50	Eriodictyol	440735
51	Evodiamine	442088
52	Ficusal	10496641
53	Fisetin	5281614
54	Galloflavin	135483971
55	Genistin	5281377
56	Glomeratose A	11972358
57	Glycitin	187808
58	Gossypol	3503
59	Hispidulin	5281628
60	Hyperoside	5281643
61	Iridin	5281777
62	Irigenin	5464170
63	Irisflorentin	170569
64	Iristectorin A	11968629
65	Isopropyl butyrate	61184
66	Isoquercetin	5280804
67	Isovanillin	12127
68	Jatrorrhizine	72323
69	Kaempferol	5280863
70	Lanosterol	246983
71	Lapachol	3884
72	Lignans	443013
73	Linoleic acid	5280450
74	Lomofungin	135426834
75	Lupeol	259846

76	Luteolin	5280445
77	Luteolin-7-O-β-D-glucoside	5280637
78	Matrine	91466
79	Methyl hexadecanoate	10378565
80	Myristic acid	11005
81	Neoeriocitrin	114627
82	Nortangeretin	96506
83	Octanoic acid	379
84	Oleanolic acid	10494
85	Oleic acid	445639
86	Oroxindin	3084961
87	Oroxylin A	5320315
88	Oxymatrine	24864132
89	Pachymic acid	5484385
90	Palmitic acid	985
91	Papaverine	4680
92	Pentagalloylglucose	65238
93	Pinocembrin	68071
94	Pinosylvin	5280457
95	Prunetin	5281804
96	Quercetin	5280343
97	Quercetin 7-O-β-D-glucoside	5381351
98	Rengyol	363707
99	Rutaecarpine	65752
100	Salicylic acid	338
101	Scutellarin	185617
102	Sibiricose A5	6326020
103	Sitogluside	5742590
104	Stigmast-7-en-3-ol	3080632
105	Tectoridin	5281810
106	Tectorigenin	5281811
107	Tectorigenin-7-O-xylosylglucoside	100968221
108	Tenuifoliside B	10055215
109	Tenuifoliside C	11968391
110	Trametenolic acid	12309443
111	Triterpenoid	451674
112	Urolithin M6	101461104
113	Ursolic acid	64945
114	Vanillil	226343
115	Zarzissine	6400641

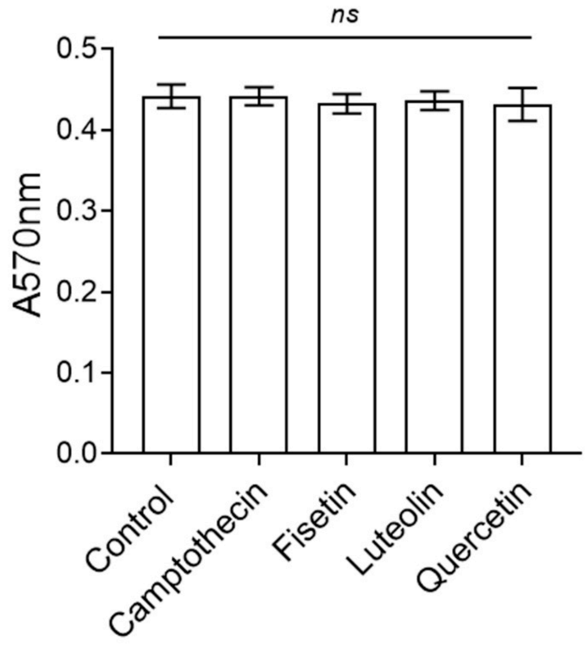

Supplementary Figure S1. The assessment of the four most promising LDHB inhibitor candidates, identified through in silico analysis and drug-likeness evaluations, on the formation of blue-purple formazan. Each compound was tested at a concentration of 100 μM and mixed with 10 nmol of NADH in a final volume of 10 μL within a 96-well plate. The reactions were conducted in 90 μL of 50 mM CHES buffer (pH 9.6) supplemented with 150 mM NaCl, 300 μM NBT, 30 μM PMS, and 0.13% gelatin. Control wells lacked inhibitors. After incubation for 5 minutes at 25 °C, absorbance at 570 nm was measured. Statistical analysis using one-way ANOVA followed by Dunnett's test revealed no interference by any compound in forming the blue-purple formazan derivative. Statistical significance was set at $p < 0.05$, with '*n.s.*' indicating results were not significant.

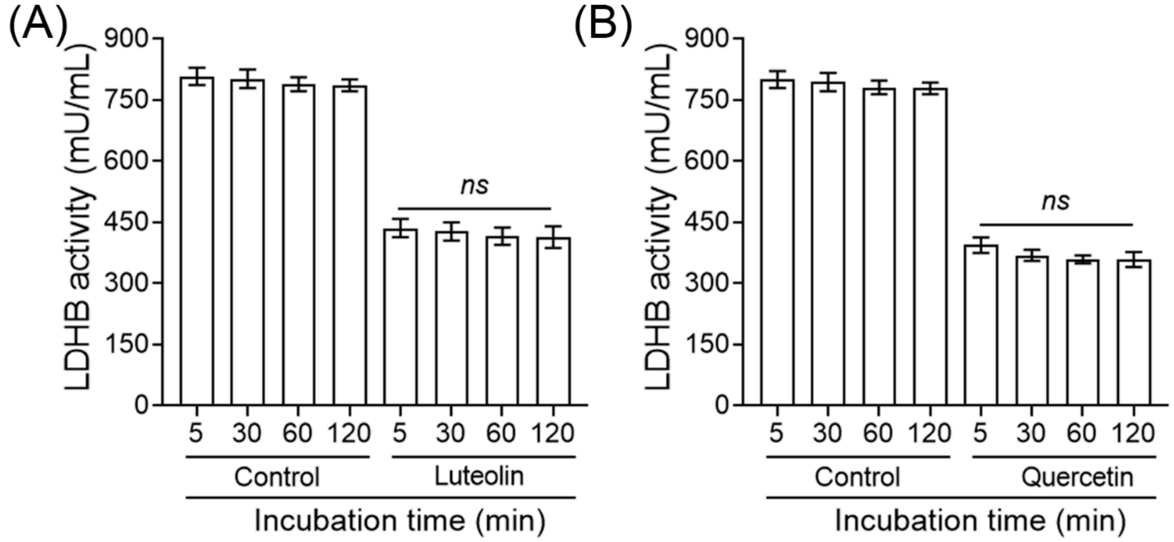

Supplementary Figure S2. The effect of pre-incubation time on the inhibitory activity of luteolin (A) and quercetin (B) on LDHB activity. Each compound (50 μM) was incubated with 2.5 nmol of LDHB in a 10 μL reaction within a 96-well plate for varying times. The reaction was carried out in 80 μL of 50 mM CHES buffer (pH 9.6) containing 150 mM NaCl, 300 μM NBT, 30 μM PMS, and 0.13% gelatin, with 10 μL of substrate mix (10 mM NAD^+ and 250 mM sodium lactate) added to initiate the reaction. LDHB activity was measured as described in the text, with control wells lacking inhibitors. Statistical significance was determined using one way ANONA ($p < 0.05$), with '*n.s.*' indicating non-significant results.
